# Personalized computational electro-mechanics simulations to optimize cardiac resynchronization therapy

**DOI:** 10.1007/s10237-024-01878-8

**Published:** 2024-08-27

**Authors:** Emilia Capuano, Francesco Regazzoni, Massimiliano Maines, Silvia Fornara, Vanessa Locatelli, Domenico Catanzariti, Simone Stella, Fabio Nobile, Maurizio Del Greco, Christian Vergara

**Affiliations:** 1 MOX, Dipartimento di Mathematica, Politecnico di Milano, Piazza Leonardo da Vinci, 32, 201333 Milan, Italy; 2 Cardiology department, S.M. del Carmine Hospital, APSS, Corso Verona, 4, Rovereto, 38068 Trento Italy; 3 LABS, Dipartimento di Chimica, Materiali e Ingegneria Chimica, Politecnico di Milano, Piazza Leonardo da Vinci, 32, 201333 Milan, Italy; 4 Institute of Mathematics, École Polytechnique Fédérale de Lausanne, Station 8, Av. Piccard, CH-1015 Lausanne, Switzerland

**Keywords:** Cardiac resynchronization therapy, Electro-mechanics simulations, Electro-anatomical mapping, Epicardial veins

## Abstract

In this study, we present a computational framework designed to evaluate virtual scenarios of cardiac resynchronization therapy (CRT) and compare their effectiveness based on relevant clinical biomarkers. Our approach involves electro-mechanical numerical simulations personalized, for patients with left bundle branch block, by means of a calibration obtained using data from Electro-Anatomical Mapping System (EAMS) measures acquired by cardiologists during the CRT procedure, as well as ventricular pressures and volumes, both obtained pre-implantation. We validate the calibration by using EAMS data coming from right pacing conditions. Three patients with fibrosis and three without are considered to explore various conditions. Our virtual scenarios consist of personalized numerical experiments, incorporating different positions of the left electrode along reconstructed epicardial veins; different locations of the right electrode; different ventriculo-ventricular delays. The aim is to offer a comprehensive tool capable of optimizing CRT efficiency for individual patients. We provide preliminary answers on optimal electrode placement and delay, by computing some relevant biomarkers such as $dP/dt_{max}$, ejection fraction, stroke work. From our numerical experiments, we found that the latest activated segment during sinus rhythm is an effective choice for the non-fibrotic cases for the location of the left electrode. Also, our results showed that the activation of the right electrode before the left one seems to improve the CRT performance for the non-fibrotic cases. Last, we found that the CRT performance seems to improve by positioning the right electrode halfway between the base and the apex. This work is on the line of computational works for the study of CRT and introduces new features in the field, such as the presence of the epicardial veins and the movement of the right electrode. All these studies from the different research groups can in future synergistically flow together in the development of a tool which clinicians could use during the procedure to have quantitative information about the patient’s propagation in different scenarios.

## Introduction

Cardiac resynchronization therapy (CRT) is a clinical treatment employed by cardiologists to treat heart failure patients affected by dyssynchronous ventricular contraction (Abraham et al. 2002), due e.g. to Left Bundle Branch Block (LBBB), an electrical conduction defect causing a late activation of the Left Ventricle (LV), and resulting in a lowered ejection fraction. The aim of CRT is to restore a coordinated ventricular motion, therefore improving systolic function and cardiac output (Kirk and Kass 2013; Delgado and Bax 2011).

The functioning of CRT relies on an implantable device called bi-ventricular pacemaker that provides electrical stimuli to the heart chambers, through the insertion of electrodes, in order to restore the synchrony and a physiological contraction of the cardiac muscle.

Usually, CRT is performed by locating one electrode in the epicardial veins of the left ventricle and another one in the right ventricle at the level of the apex. Often, the two stimuli are characterized by a delay in order to improve CRT performance. Clinicians, at the moment of the procedure, need to select suitable electrodes locations and an effective value of the stimuli delay. There is no global agreement on the optimal procedure settings ensuring a success of the therapy and long-term benefit for the patients (Delgado et al. 2011; Lee et al. 2017). Indeed, $30 \%$ of patients do not respond to CRT, as several clinical trials show, like MIRACLE (Sutton et al. 2003). More recently, discussion has begun on the concept of non-responders as opposed to that of negative responders and non-progressors. In any case, the number of patients whose clinical status does not improve is between 20% and 30%, see the recent review Lu et al. (2022) for further details. For such reasons CRT still needs to be improved.

In this context, mathematical and computational cardiac models may support clinicians by enabling the virtual reproduction of the heart function under different working conditions, avoiding to resort to invasive and expensive procedures. Indeed, mathematical models describing the physics of the heart, both in physiological and pathological conditions, are becoming a powerful tool to support therapeutic planning for several cardiac disorders, i.e. arrhythmias, cardiomyopathies, myocardial infarction, heart failure (Relan et al. 2011; Chan et al. 2012), and, as in the present case, LBBB and ventricular dyssynchrony (Kerckhoffs et al. 2009; Aguado-Sierra et al. 2011).

Cardiac models may in general account for electrical propagation, mechanical contraction and hemodynamics (Quarteroni et al. 2017). In view of CRT simulation and optimization, several computational studies have been performed by different research groups (see the review by Lee et al. (2023) and Table 1). Some of them focused only on electrophysiology, often employing patient-specific ECG data and guaranteeing high efficiency; important outcomes from the electrical point of view could be obtained, for example the influence of conduction velocity system and of pacing close to scars on the CRT success; however such studies neglect any indication about the mechanical restoring. Other studies relied on Electro-Mechanics (EM) models, possibly personalized by means of electrical (mapping or ECG) or pressure-volume measures; in some cases patient’s fibrosis has been accounted for. In Table 1, columns “EP Calibration” and “MEC Calibration” indicate whether electrophysiology and mechanics models, respectively, have been personalized (i.e. calibrated) by means of available clinical data ($\checkmark$) or performed using representative average values ($\times$).

**Table 1:** State of the art of some representative computational studies for simulating CRT. In the bottom part we highlighted those works that included some preliminary investigations about CRT optimization. LV (Left Ventricle), RV (Right Ventricle), RA (Right Atrium), EM (Electro-Mechanics), EP (Electro-Physiology), MEC (Mechanics), Opt CRT (Optimization of Cardiac Resynchronization Therapy)

Work	Human	Fibrosis	EM	EP	MEC	Opt	RV	Epicardial	Closed	RA
	LV			Calibr.	Calibr.	CRT		Veins	LPM	pacing
										preload
Kerckhoffs et al. (2003)	$\times$	$\times$	$\checkmark$	$\times$	$\times$	$\times$	$\times$	$\times$	$\times$	$\times$
Kerckhoffs et al. (2009)	$\checkmark ^\dagger$	$\times$	$\checkmark$	$\times$	$\times$	$\times$	$\checkmark$	$\times$	$\times$	$\times$
Sermesant et al. (2012)	$\checkmark$	$\checkmark$	$\checkmark$	$\checkmark ^+$	$\checkmark$	$\times$	$\checkmark$	$\times$	$\times$	$\times$
Hyde et al. (2015)	$\checkmark$	$\times$	$\times$	$\times$	$\times$	$\times$	$\checkmark$	$\times$	$\times$	$\times$
Kayvanpour et al. (2015)	$\checkmark$	$\times$	$\checkmark$	$\checkmark ^{*}$	$\times$	$\times$	$\checkmark$	$\times$	$\times$	$\times$
Crozier et al. (2016)	$\checkmark$	$\times$	$\checkmark$	$\checkmark ^*$	$\checkmark$	$\times$	$\checkmark$	$\times$	$\times$	$\times$
Costa et al. (2020)	$\checkmark$	$\checkmark$	$\times$	$\times$	$\times$	$\times$	$\times$	$\times$	$\times$	$\times$
Reumann et al. (2007)	$\checkmark$	$\times$	$\times$	$\times$	$\times$	$\checkmark$	$\checkmark$	$\times$	$\times$	$\times$
Villongco et al. (2016)	$\checkmark$	$\checkmark$	$\times$	$\checkmark ^*$	$\times$	$\checkmark$	$\checkmark$	$\times$	$\times$	$\times$
Pluijmert et al. (2016)	$\times$	$\times$	$\checkmark$	$\times$	$\times$	$\checkmark$	$\checkmark$	$\times$	$\checkmark$	$\times$
Lee et al. (2017)	$\checkmark$	$\checkmark$	$\checkmark$	$\checkmark ^{*}$	$\checkmark$	$\checkmark$	$\times$	$\times$	$\times$	$\times$
Isotani et al. (2020)	$\checkmark$	$\checkmark$	$\checkmark$	$\times$	$\checkmark$	$\checkmark$	$\checkmark$	$\times$	$\checkmark$	$\times$
Albatat et al. (2021)	$\checkmark$	$\checkmark$	$\times$	$\times$	$\times$	$\checkmark$	$\checkmark$	$\times$	$\times$	$\times$
Fan et al. (2022)	$\times$	$\checkmark$	$\checkmark$	$\times$	$\checkmark$	$\checkmark$	$\times$	$\times$	$\checkmark$	$\checkmark$
Present work	$\checkmark$	$\checkmark$	$\checkmark$	$\checkmark$	$\checkmark$	$\checkmark$	$\times$	$\checkmark$	$\times$	$\times$

$^\dagger$ canine LV has been used

$^+$ EP calibration performed by means of anatomical electrical mapping

$^*$ EP calibration performed by means of ECG measures

More recently, some work performed a virtual CRT optimization by varying the location of the electrodes and the stimuli delay. All the studies confirmed that the best left electrode location is the left ventricle lateral wall (Pluijmert et al. 2016; Lee et al. 2017; Isotani et al. 2020; Fan et al. 2022); in Albatat et al. (2021) the authors stressed in particular the importance of stimulating with the left electrode the latest activation region, whereas in Villongco et al. (2016) that the left pacing should slightly anticipate the right one. However, in all the cases the positioning of electrodes has not taken into account anatomical constraints such as coronary sinus and left ventricle epicardial veins. Moreover, only the acute scenario, i.e. what happens just after the device implantation, has been analysed.

In this work, a patient-specific EM computational model (Stella et al. 2022) is employed to study different CRT scenarios in acute conditions by virtually moving the left electrode along the patient-specific coronary sinus and epicardial veins, as happens in the clinical setting. Moreover, we consider different right electrode locations and stimulation delays. For each of the analysed patients we personalized the model electrical properties by using Electro-Anatomical Mappings (EAM), as proposed in Stella et al. (2020); Vergara et al. (2022), and mechanical properties by using volume and pressure measures. Notice that EAM acquisition is obtained by cardiologists during the CRT procedure and no additional invasive mapping is required for our purposes. At the best of our knowledge, this is the first computational study that exploits patient-specific anatomically compatible locations of the left electrode in CRT studies, i.e. allowing pacing locations identified from the patient coronary sinus/epicardial veins anatomy. Moreover, we used a personalized EM model for CRT optimization and we explore, for the first time, different right electrode positions.

Six real cases of patients implanted with a CRT device are presented, all affected by LBBB and all treated at the S. Maria del Carmine Hospital in Rovereto (TN), Italy, by using the Latest Electrically Activated Segment (LEAS) in the epicardial veins to locate the left electrode. Pre-operative electrical and mechanical clinical measurements have been employed to personalize the Eikonal-Reaction-Mechanics (ERM) model (Neic et al. 2017; Stella et al. 2022) on each patient, whereas electrical measures obtained by an intermediate right pacing, carried out during the CRT implantation procedure, have been used to validate our model.

The idea is to use the personalized ERM model to provide useful information about the optimal CRT delivery for the patient, by comparing activation and contraction of different virtual configurations just after the implantation of the device. This is justified by assuming that the hemodynamic and electrical properties characterizing the patient do not change immediately after the beginning of CRT stimulation. The beneficial effects of every CRT scenario are evaluated and compared using suitable mechanical biomarkers that are used in clinical practice to evaluate acute outcomes of the therapy (Sermesant et al. 2012; Zanon et al. 2015). Such quantities are able to provide information about the restoring of the heart function, such as the Ejection Fraction (EF) and the maximum rate of pressure change, that can be easily computed as post-processing of the numerical ERM results.

## Materials and methods

### Overview of the work

The overall flowchart of the method we developed to perform virtual CRT scenarios and compare their outcomes is mainly composed by three major steps:

- Pre-processing of the data in order to obtain the computational meshes and the activation times provided by an Electro-Anatomical Mapping System (EAMS) obtained by cardiologists during the CRT procedure (no additional invasive mapping is required);
- Personalization of the Eikonal-Reaction-Mechanics model in terms of both electrical and mechanical parameters, in sinus rhythm (pre-operative) conditions;
- Simulation of CRT virtual scenarios by varying left and right electrodes positions and delay.

**Fig. 1:**
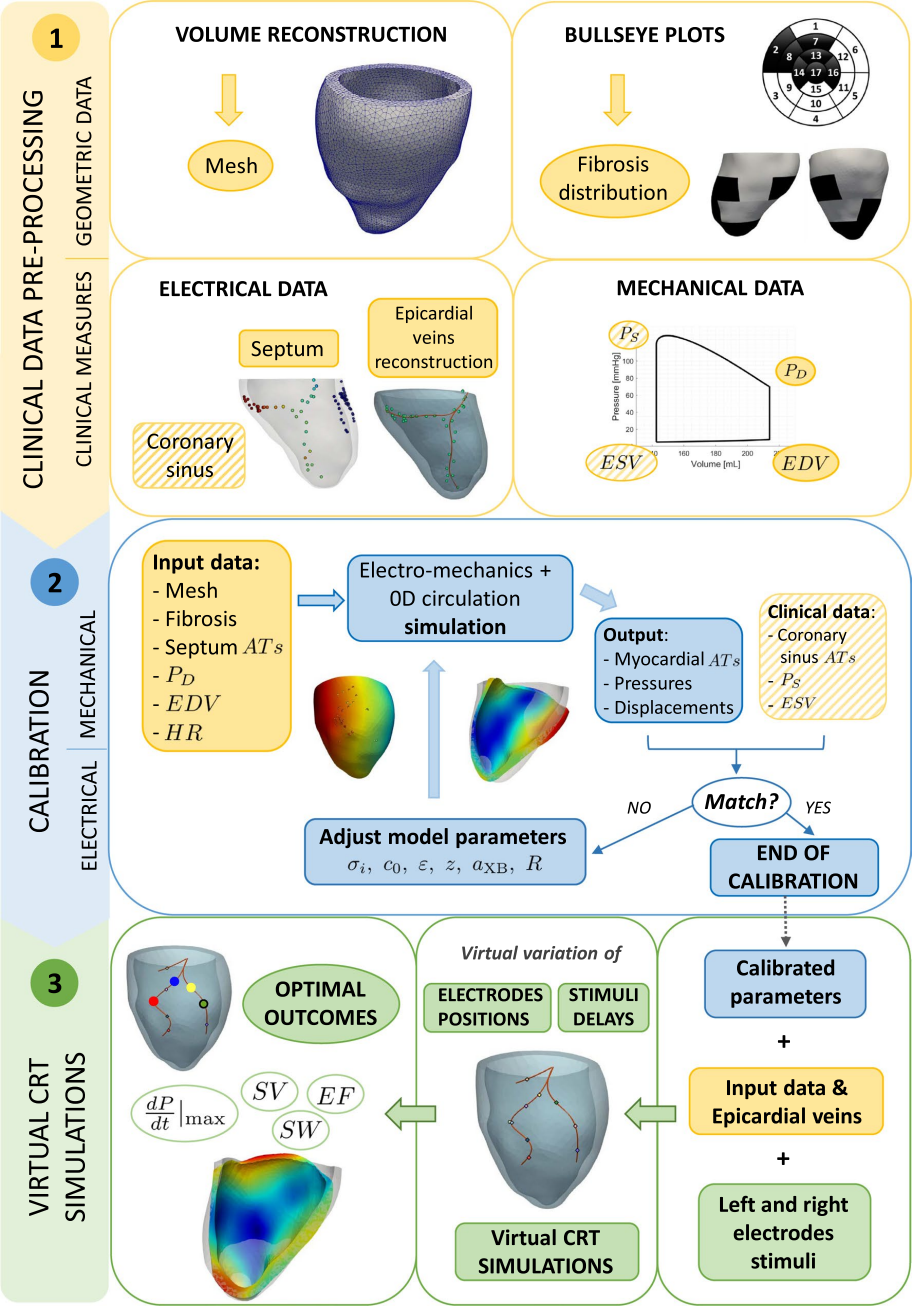
Pipeline of the overall method. Top, yellow sector: Pre-processing procedures to obtain the input data; Middle, blue sector: Electrical and mechanical calibrations to obtain the personalized parameters; Bottom, green sector: Virtual CRT simulations. AT stands for Activation Times

In Fig. 1 we report the complete flowchart of the steps of our strategy that will be detailed in the following paragraphs.

### Clinical data

We have at disposal six patients (referred in what follows as P2, P3, P4, P6, P8, P11, in accordance with previous numbering taken from Stella (2021)) who underwent CRT at Santa Maria del Carmine Hospital, Rovereto (TN), Italy. All the patients suffered from ventricle dyssynchrony caused by LBBB. Patients P6, P8, P11 presented fibrotic regions. See Table 2a for demographic data.

**Fig. 2:**
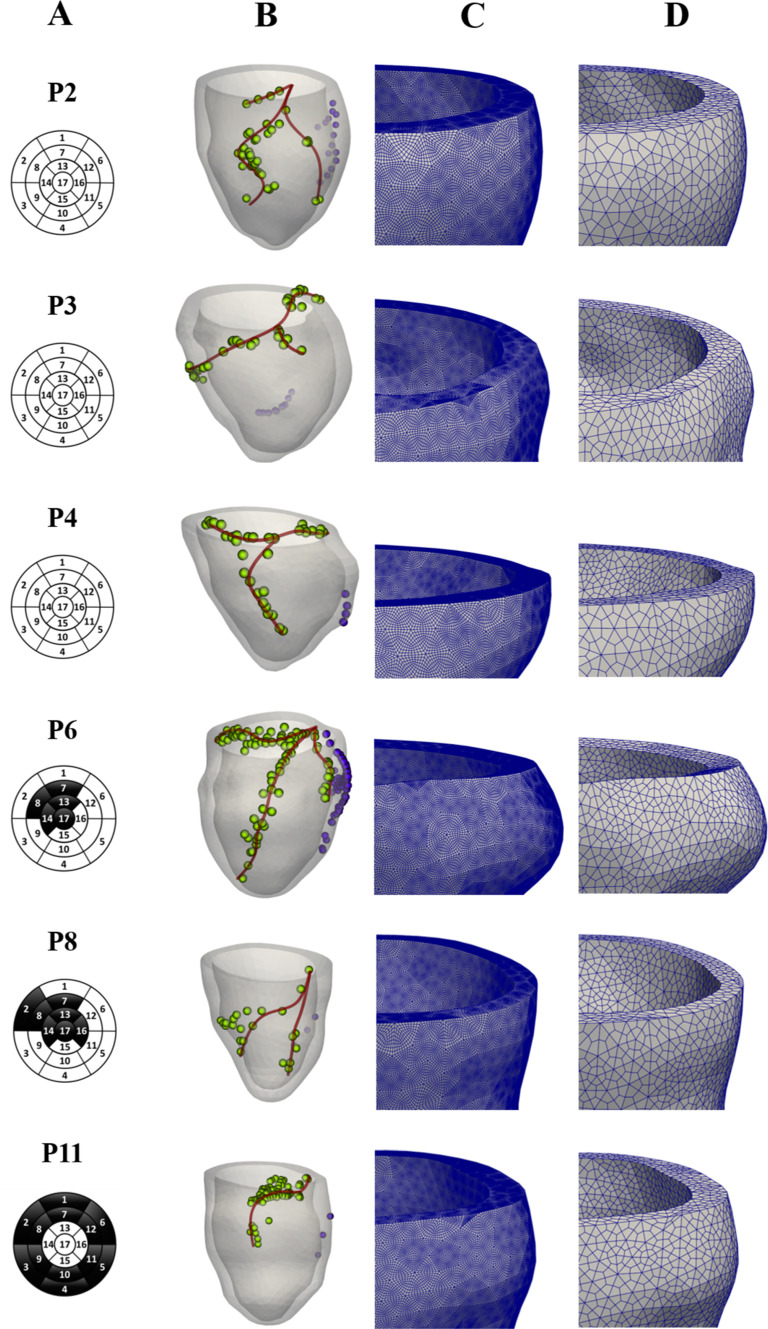
From the left: A) 17-level segments bullseye plots of the fibrotic regions; B) Patient’s reconstructed LV and epicardial veins geometries, together with points of EAMS measures. In green the measure points used for the veins’ reconstruction; C) Zoom into the fine mesh for electrophysiology simulations; D) Zoom into the coarse mesh for mechanics simulations

For all the patients we had at disposal different sets of data:

- MRI images used for the segmentation and reconstruction of the left ventricle geometry. These have been acquired at diastasis, i.e. when the mitral valve partially closes and the atrial contraction starts. To recover the end diastolic configuration (according to the patient’s measure EDV, see Table 2b below) used as starting point for the isovolumic contraction, we perform a preliminary numerical inflation (depending on the patient, in the range $[4\%-9\%]$). This allowed us to build the computational meshes (see Fig. 2) for the numerical simulations (see Stella et al. (2020) for further details);
- Bullseye diagram from late gadolinium enhancement-MRI (LGE-MRI), highlighting the fibrotic regions distribution in terms of the 17-segment division of the LV myocardium. See Fig. 2;
- Electrical activation times obtained during sinus rhythm (pre-operative scenario) with the contact ${EnSite Precision}^{{TM}}$ EAMS (Eitel et al. 2010), whose utility was threefold. First, measurements of activation times in the septal region, when available (i.e. for P2,P3,P4,P6), were used as input to the electrical propagation model in pre-operative (sinus rhythm) conditions. Secondly, measurements at the coronary sinus were used to calibrate electrical conductivities (Vergara et al. 2022). See Table 2b. Finally, the spatial distribution of acquisition points along the lateral LV epicardium was used to identify the epicardial veins geometry (see Fig. 2 and (Stella et al. 2020) for further details). Notice that at S. Maria del Carmine Hospital in Rovereto (TN) cardiologists always perform EAMS during the CRT procedure to identify the latest activation region and place the left electrode. Thus, no additional invasive mapping is required for our purposes;
- Electrical activation times obtained during right pacing (only right electrode active) with EAMS. These data are acquired by cardiologists as a preliminary analysis before CRT implantation and will be used in this study to validate the electrical calibration obtained using pre-operative data. See Table 2b for further details.
- Measures of the End Systolic and Diastolic Volumes (ESV and EDV) obtained by MRI (with corresponding Stroke Volume (SV) and Ejection Fraction (EF)), and of Diastolic and Systolic Pressure values (denoted as $P_{D}$ and $P_{S}$) measured at the arm, together with the Heart Rate (HR). See Table 2c.

**Table 2:** Patients’ clinical data acquired at S. Maria del Carmine Hospital (Rovereto, TN) between July 2014 and September 2015. (2a) Patients’ demographic data and cardiac conditions. (2b) Patients’ electrical data acquired in pre-operative conditions and during the right-pacing procedure. Activation times ranges for acquisitions at the coronary sinus are reported. (2)c Patient’s data regarding cardiac volumes, pressures and heart rate

(a) Demographic data.							
	Age	Sex	Fibrosis	NYHA			
P2	65	M	No	III			
P3	74	F	No	II			
P4	62	F	No	III			
P6	64	F	Yes	III			
P8	84	M	Yes	III			
P11	64	M	Yes	III			

(b) Clinical data used for electrophysiology model calibration and validation. $^*$ means that no data are available and that 3 stimulation points are selected a priori, see Sect. 2.5.1.							
	Pre-operative			Right Pacing			
	Septum	Coronary sinus		Coronary sinus			
	$\#$data	$\#$data	[$AT_{\text {min}}$, $AT_{\text {max}}$] [s]	$\#$data	[$AT_{\text {min}}$, $AT_{\text {max}}$] [s]		
P2	15	52	[0.07, 0.12]	45	[0.04, 0.15]		
P3	9	37	[0.11, 0.14]	16	[0.12, 0.19]		
P4	4	31	[0.08, 0.15]	15	[0.14, 0.18]		
P6	61	84	[0.07, 0.13]	23	[0.11, 0.17]		
P8	3*	17	[0.06, 0.14]	58	[0.11, 0.14]		
P11	3*	31	[0.06, 0.16]	-	-		

(c) Clinical data used for mechanical and Windkessel models calibration.							
	${ {ESV}}$	${ {EDV}}$	${ {SV}}$	${ {EF}}$	${ {P}}_{{ {D}}}$	${ {P}}_{{ {S}}}$	${ {HR}}$
	$[\textrm{ml}]$	$[\textrm{ml}]$	$[\textrm{ml}]$	$[\%]$	$[\textrm{mmHg}]$	$[\textrm{mmHg}]$	$[\textrm{bpm}]$
P2	425	501	76	15.2	60	110	85
P3	238	350	112	32.0	75	140	75
P4	226	300	73	24.5	80	120	62
P6	236	318	82	25.8	70	110	76
P8	143	214	71	33.2	70	130	68
P11	173	231	58	25.1	80	110	110

Notice that, following Stella et al. (2020), an alignment procedure is employed in order to merge geometric (computational meshes) and electrical (EAM) data. Ensuring compatibility between the two different reference systems is indeed necessary. In particular, the procedure consists of three steps: (i) reference points selection (three couples of points taken from the electrical and geometric data); (ii) geometric alignment (rotation and translation of the point cloud of electrical data to match the reconstructed LV surface); (iii) Nearest Neighbour Search projection (movement of each cloud point to coincide with the geometric nearest one). See Stella et al. (2020) for further details.

Finally, we notice that we have at disposal also CT images for another case (P12), which are used for a qualitative validation of the epicardial veins reconstruction. Starting from these images, we reconstruct the coronary veins applying in the vmtk software the *vesselness* filter provided in Frangi et al. (1998) as a pre-processing step for a better visualization of the lumen of the vessel. We then performed a level set segmentation after a colliding fronts initialization (Antiga et al. 2008).

### Mathematical model

For the computational model we used the Eikonal-Reaction-Mechanics (ERM) model proposed in Stella et al. (2022), so that we refer the interested reader there for details. Here, it is enough to report the coupled models and the relevant parameters for the calibration:

1. An offline Eikonal-Diffusion model (Colli-Franzone et al. 2014) to determine activation times $\psi$ in electrophysiology, see in particular Stella et al. (2022). This is characterized by parameter $c_0$ that tunes the velocity of the depolarization wave along the fibre direction for a planar wavefront, by the dimensionless parameter $\varepsilon$ that regulated the impact of the wavefront curvature on its propagation velocity, and by the conductivity tensor normalized with respect to the surface-to-volume ratio and the transmembrane capacitance (see Stella et al. (2022)):
  1 $$\begin{aligned} \widehat{{\textbf {D}}} = z \widehat{\sigma }_s \varvec{1} + z ({\widehat{\sigma }}_f - {\widehat{\sigma }}_s)\varvec{f} \otimes \varvec{f} + z({\widehat{\sigma }}_n - {\widehat{\sigma }}_s)\varvec{n} \otimes \varvec{n}. \end{aligned}$$
  Vectors $\varvec{f}$, $\varvec{s}$ and $\varvec{n}$ are the fibres, sheets and normal directions, $\{{\widehat{\sigma }}_{i}\}_{i\in \{f,s,n\}}$ are the respective conductivities and $z \in [0,1]$ is a parameter suitably calibrated to consider the decreased conductivity in fibrotic regions (with $z = 1$ denoting healthy tissue);
2. The solution $\psi$ obtained at step 1 is in turn used to shift the calcium transient precomputed by solving an offline Reaction problem (Neic et al. 2017) for the intracellular calcium concentration $[\textrm{Ca}^{2+}]_i$, namely a simplified version of the monodomain equation. The latter consists of a single-cell simulation in which we surrogate the diffusive currents with a forcing term that triggers the action potential, coupled to the *ToR-ORd* ionic model (Tomek et al. 2019);
3. The temporal loop where at each time step we have the coupling among:
  - The Mechanical Activation (MA) provided by the *RDQ20-MF* model (Regazzoni et al. 2020), characterized by *cross-bridge stiffness* $a_{\text {XB}}$, which quantifies the myocardial tissue contractility. This allows to compute the active tension $T_a$;
  - The Active-Passive Mechanics (APM); in particular, we used the *Usyk constitutive law* (Usyk et al. 2002), with a weak incompressibility penalization term $\frac{K}{2} (J - 1) log(J)$ (*J* being the variation of volume and *K* the penalty parameter), for the passive mechanics and the active stress obtained by assuming that $T_a$ acts only in the fibres direction $\varvec{f}$ (Nash and Panfilov 2004). In order to account for the tissue stiffening and reduced contractility in fibrotic regions (see, e.g. Holmes et al. (1997); Sirry et al. (2016)), we rescale the material law stiffness by a factor $[z +(1-z)4.56]$ (according to Salvador et al. (2022) and the experiments in Sack et al. (2018), where the authors estimated the ratio between the stiffness in presence of scar and in the healthy case), and the active contractility by a factor *z* (being the *z* the factor introduced at point 1);
  - A two-element Windkessel (W) model for the circulatory system during the ejection phase (Westerhof et al. 2009; Quarteroni et al. 2016), characterized by the resistance *R* and the compliance *C*, or a linear ramp for the pressure during the filling phase.
  Specifically, MA provides the active tension for APM, whereas the latter allows us to prescribe the mechanical deformation to the former. APM and W are coupled during the ejection phase, through the exchange of LV pressure and volume, whereas during the filling the pressure data of the prescribed ramp are assigned to APM. On the other hand, isovolumetric phases have been managed by interpreting the 0D pressures as Lagrange multipliers to enforce the volume conservation constraint. To account for the pericardium APM model was equipped by Robin-like boundary conditions (Pfaller et al. 2018).

**Fig. 3:**
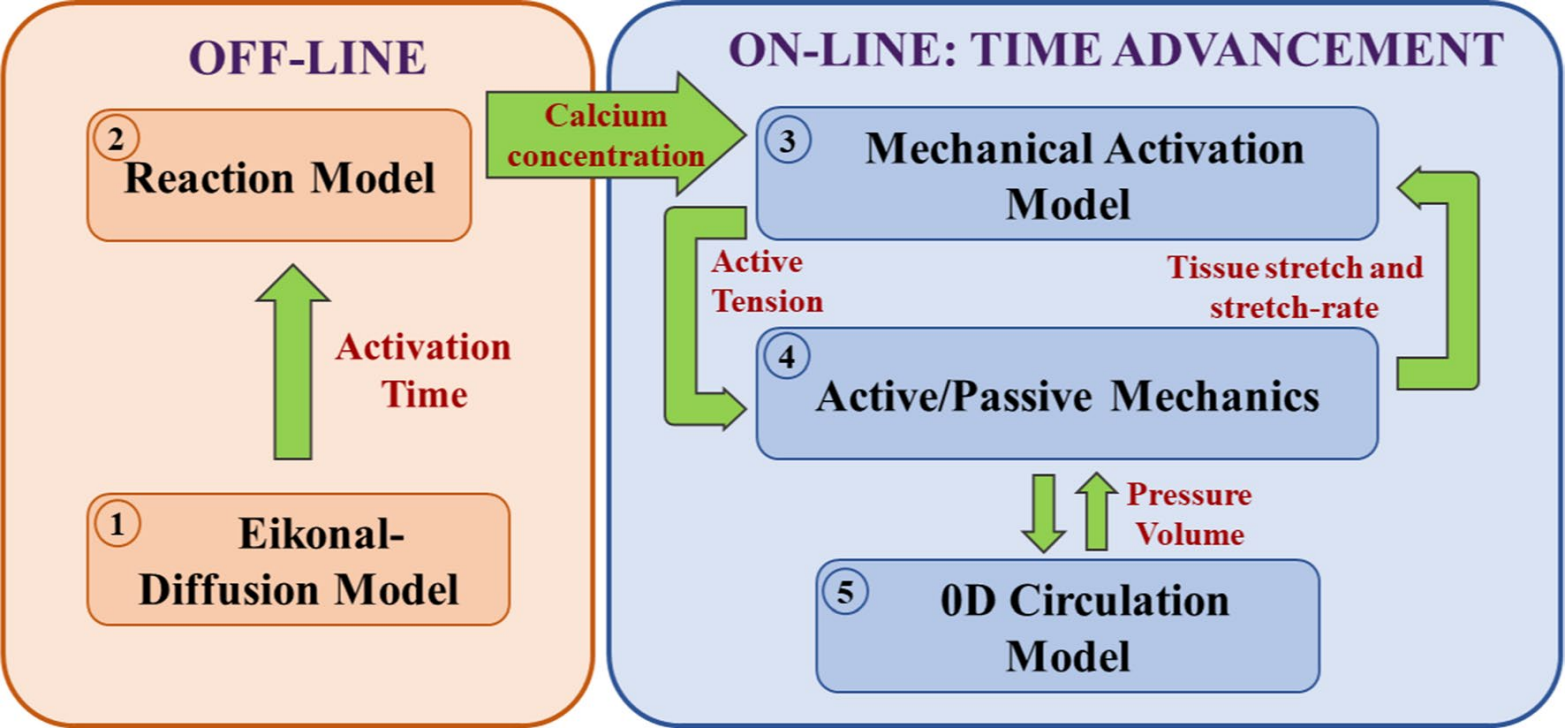
Structure of the Eikonal-Reaction-Mechanics model, highlighting the couplings among the different cores

Notice that the electrophysiology problem (steps i and ii) is based on computing the activation times $\psi (\varvec{x})$ with an Eikonal model and then to localize in the Reaction problem an applied current for each point $\varvec{x}$ in a temporal neighbourhood of $\psi (\varvec{x})$. This allowed us to surrogate the diffusion process (Stella et al. 2022).

Electrical propagation and mechanical contraction are highly influenced by cardiac fibres’ direction. Since standard imaging techniques do not provide geometric information, we used here the Laplace-Dirichlet rule-based algorithm described in Bayer et al. (2012); Piersanti et al. (2021). In particular, we employed the following angles as boundary conditions: $60^o$ for fibres at the epicardium, $60^o$ for fibres at endocardium, $20^o$ for sheets on epicardium and $20^o$ for sheets on endocardium. See also Stella et al. (2020) for further details.

We highlight also that our model is based on the simplifying assumption of neglecting the *mechano-electrical feedbacks*, allowing to solve electrophysiology (step i and ii) outside of the temporal loop. The solution of the Reaction model (step ii) is obtained off-line for each heartbeat duration and evaluated in each mesh point.

The choice of using a 0D model for blood fluid dynamics is justified by the major interest of the present work in the electrical and mechanical contraction of the cardiac muscle, rather than blood dynamics in the chamber.

In Fig. 3 we report a comprehensive picture of the computational model.

### Numerical approximation

All computational methods for the numerical approximation of the ERM mathematical model are implemented in the high-performance C++ library life$^{\texttt {x}}$, developed at MOX, Dipartimento di Matematica, with the collaboration of LaBS, Dipartimento di Chimica, Materiali e Ingegneria Chimica (both at Politecnico di Milano)^1^ (Africa 2022; Africa et al. 2023).

For the numerical solution of time-dependent problem (iii) in Sect. 2.3, we consider a segregated method, based on a loosely coupled strategy for mechanics couplings with both MA and 0D model (Stella et al. 2022). We discretize all the evolutionary subproblems by means of the BDF1 scheme using a time step $\Delta t = {1e-4\,\mathrm{\text {s}}}$. MA problem, which is instead approximated by the Forward Euler method, requires a finer time step for numerical stability purposes. Hence, it is advanced resorting to an inner iteration loop with $\Delta t = {2.5e-5\,\mathrm{\text {s}}}$ (see Stella et al. (2022)).

Each subproblem is discretized in space using the Finite Element Method (FEM) of order 1 on hexahedral meshes (Q1). Two nested meshes are generated: a coarser mesh for mechanics ($h \simeq {3.5\,\mathrm{\text {m}\text {m}}}$) and a finer one for electrophysiology ($h \simeq {0.8\,\mathrm{\text {m}\text {m}}}$) obtained by recursively splitting each element of the coarser one. See Fig. 2.

We find that five cardiac cycles were enough to reach a limit cycle for our ERM model. Accordingly, for each case we simulate five cardiac cycles (length of period depending on the patient’s measured cardiac frequency) and we analyse the results of the fifth cycle only.

### Personalization of the model

To calibrate the model in pre-operative conditions, we proceed in two steps, considering first the electrophysiology and then the mechanics, due to the absence of feedback from the latter to the former. The values of all the other parameters used in the numerical experiments are reported in Table 3.

**Table 3:** Non-calibrated parameters used in the numerical simulations: Mechanical Activation parameters taken from Regazzoni et al. (2020), Passive Mechanics parameters taken from Usyk et al. (2002), Pericardium and Windkessel parameters taken from Stella et al. (2022)

	$SL_0 \ [{\upmu \text {m}}]$	$\bar{k}_d \ [{\upmu M}]$	$\alpha _{k_d} \ [{\upmu M \upmu ^{-1}\text {m}}]$	$\gamma \ [-]$
Mechanical	2.2	0.4	$-0.2083$	30
activation	$k_{\text {off}} \ [{\text {s}^{-1}}]$	$k_{\text {basic}} \ [{\text {s}^{-1}}]$	$\mu _{f_{\mathscr {P}}}^0 \ [{\text {s}^{-1}}]$	$\mu _{f_{\mathscr {P}}}^1 \ [{\text {s}^{-1}}]$
	40	8	32.255	0.768
	$b_f$ [-]	$b_s$ [-]	$b_n$ [-]	$b_{fs}$ [-]
Passive	8	6	3	12
mechanics	$b_{fn}$ [-]	$b_{sn}$ [-]	*C* [kPa]	*K* [kPa]
	3	3	0.88	50
Pericardium	$K^{\text {epi}}_{\bot } \ [{\text {Pa}\text {m}^{-1}}]$	$K^{\text {epi}}_{\Vert } \ [{\text {Pa}\text {m}^{-1}}]$	$C^{\text {epi}}_{\bot } \ [{\text {Pa}\text {s}\text {m}^{-1}}]$	$C^{\text {epi}}_{\Vert } \ [{\text {Pa}\text {s}\text {m}^{-1}}]$
	$2 \cdot 10^5$	$2 \cdot 10^4$	$2 \cdot 10^4$	$2 \cdot 10^3$
Windkessel	$\bar{p}_{\text {MVO}}^{0D} \ [{\text {mmHg}}]$	$\bar{p}_{\text {ED}}^{0D} \ [{\text {mmHg}}]$	$C \ [{\text {m}^3 \text {Pa}^{-1}}]$	
	5	10	$4.5 \cdot 10^{-9}$	

#### Electrical calibration

We prescribe as input to the Eikonal-Diffusion model the activation times measured at the septum. When these are not available (i.e. for P8 and P11), three equispaced points are selected at the septum, to surrogate the action of the Purkinje network. Specifically, we locate them on the right side of the septum mimicking the activation occurring through the Right Ventricle (RV) and not through the LV Purkinje fascicles, according to standard observations from (Strik et al. 2012).

Then, we calibrate the Eikonal-Diffusion model parameters, following the criterion of minimizing the discrepancy between the clinically measured activation times $t_i^{\text {clin}}$, representing the pre-operative scenario, on the epicardial veins and their computational counterparts $t_i^{\text {comp}}$ (calibration error):

2 $$\begin{aligned} e[\%] = \frac{1}{N T_{\max }}\sum _{i=1}^{N}| t_i^{\text {clin}} - t_i^{\text {comp}} | \cdot 100, \end{aligned}$$

where *N* is the number of activation times clinically recorded and $T_{\max }$ is the maximum activation time recorded. Due to the computational challenges linked to automatic optimization procedures with an underlying differential model, we rely on a manual procedure. Specifically, starting from baseline values (Stella et al. 2022), we undertake an iterative tuning approach consisting of the following steps. First, we calibrate the conduction velocity by controlling the scalar parameter $c_0$ to minimize the discrepancy *e*. This first step results in the major decrease of the error along the calibration procedure. Subsequently, while maintaining $c_0$ constant, we fine-tune the dimensionless parameter $\varepsilon$, which governs the curvature effects on wave front propagation. This step further enhances the calibration by diminishing the remaining discrepancy *e*. In patients with fibrotic tissue, we consider a modified conductivity tensor, given by (1), where *z* is assigned 1 in healthy regions and a value less than 1 in fibrotic regions. To determine the appropriate value for *z* in fibrotic regions, we repeat the aforementioned procedure across various *z* values, selecting the one that minimizes the discrepancy *e*. As a convergence criterion, we require that the final relative error *e* is lower than $10\%$. Such a value allows us to obtain good validation errors (see Sect. 3.2 and Table 4). Thus, considering the noisy nature of the data, we believe that our tolerance choice is significant in terms of calibration accuracy.

#### Mechanical calibration

Once the electrical calibration is successfully concluded, we proceed with the calibration of the mechanical and hemodynamic parameters. Some parameters of the model are readily available as patients’ measures, namely the HR, the EDV and the aortic valve opening pressure, which we surrogate with the clinically measured diastolic arterial pressure $P_D$. The end-diastolic left ventricular pressure, needed as initial condition in the EM model, has been automatically determined as the unique pressure that yields the desired EDV. We remark that the HR is not only used in the APM model to define the heartbeat period, but it is also employed to compute the calcium transient within the ToR-ORd ionic model, which was built based on human data (Tomek et al. 2019), and is capable to reproduce the restitution curve of cytosolic calcium: the higher the heart rate, the larger the peak in calcium concentration The calcium transient is in turn provided to the RDQ20-MF model, which reproduces the force-calcium dependency: the higher the calcium concentration, the larger the apparent calcium sensitivity and force magnitude (Regazzoni et al. 2020). Thanks to the combined effect of the two above-mentioned mechanisms, our ERM model captures the inotropic effect of heart rate increase, that is to say a dependence of apparent contractility upon variations in the heart rate. See the Appendix for more details on the mechanical model behaviour.

Finally, we employ the two remaining clinical quantities available, namely ESV and $P_S$, to calibrate two parameters of the model that rule the cardiac contractility and the afterload. These parameters are the cross-bridge stiffness $a_{\text {XB}}$ and the Windkessel resistance *R*, respectively. We observe that the number of parameters left to calibrate matches the number of the available clinical quantities not yet used for the calibration. In case more clinical quantities were available, additional parameters could be calibrated on a patient-specific basis, such as the passive stiffness (see e.g. Asner et al. (2016); Dabiri et al. (2019); Kovacheva et al. (2021)). However, in order not to make the calibration procedure underdetermined, we here decided the keep the passive stiffness to its baseline value, which has not been searched by means of an optimization; rather, it has been set to a reference value. Alternatively to our procedure, one could select a priori the contractility values (different in the healthy and fibrotic regions) and use the passive stiffness as an optimization parameter.

Due to the higher computational cost of the APM model compared to the Eikonal-Diffusion model, following an iterative procedure by running the full-order APM model for any change of model parameters would entail a significant computational cost. Hence, in order to speedup this step of the calibration pipeline, we rely on the cardiac emulator proposed in Regazzoni and Quarteroni (2021). The emulator is a surrogate cardiac model, built through a data-driven approach from a few Pressure-Volume (PV) loops computed through the computational model. Once it is constructed, it allows predicting PV loops for new values of the parameters in less than one millisecond on a single-core laptop, with an approximation lower than 1%. The workflow is thus as follows:

1. Run a few cycles with an initial guess of *R* and for two different values of $a_{\text {XB}}$;
2. Construct the cardiac emulator, by relying on the Python library cardio-emulator^2^;
3. Find the optimal value of the parameters $a_{\text {XB}}$ and *R* by fitting ESV and $P_S$ with the cardiac emulator prediction (we notice that this step is computationally fast, thanks to the use of the lightweight emulator);
4. Run a 3D-0D simulation with the selected parameters, to check that the fit of clinical data is satisfactory.

### CRT scenarios under investigation

To study the optimal CRT configurations in acute conditions, the idea of this paper is to virtually vary the activation conditions of the stimulation by assuming that the electrical and mechanical conditions just after the implantation are the same of the pre-operative case. Indeed, CRT induces significant effects on the cardiac muscle only some months after the implant (Jaffe and Morin 2014).

In what follows, we refer to LEAS as the *Latest Electrically Activated Segment*, i.e. the point accessible through the reconstructed epicardial veins characterized by the highest computed activation time. LEAS is used by some cardiologists for the location of the left electrode (Del Greco et al. 2017). Notice that in Vergara et al. (2022) we proved that computational methods are able to well predict LEAS position.

**Fig. 4:**
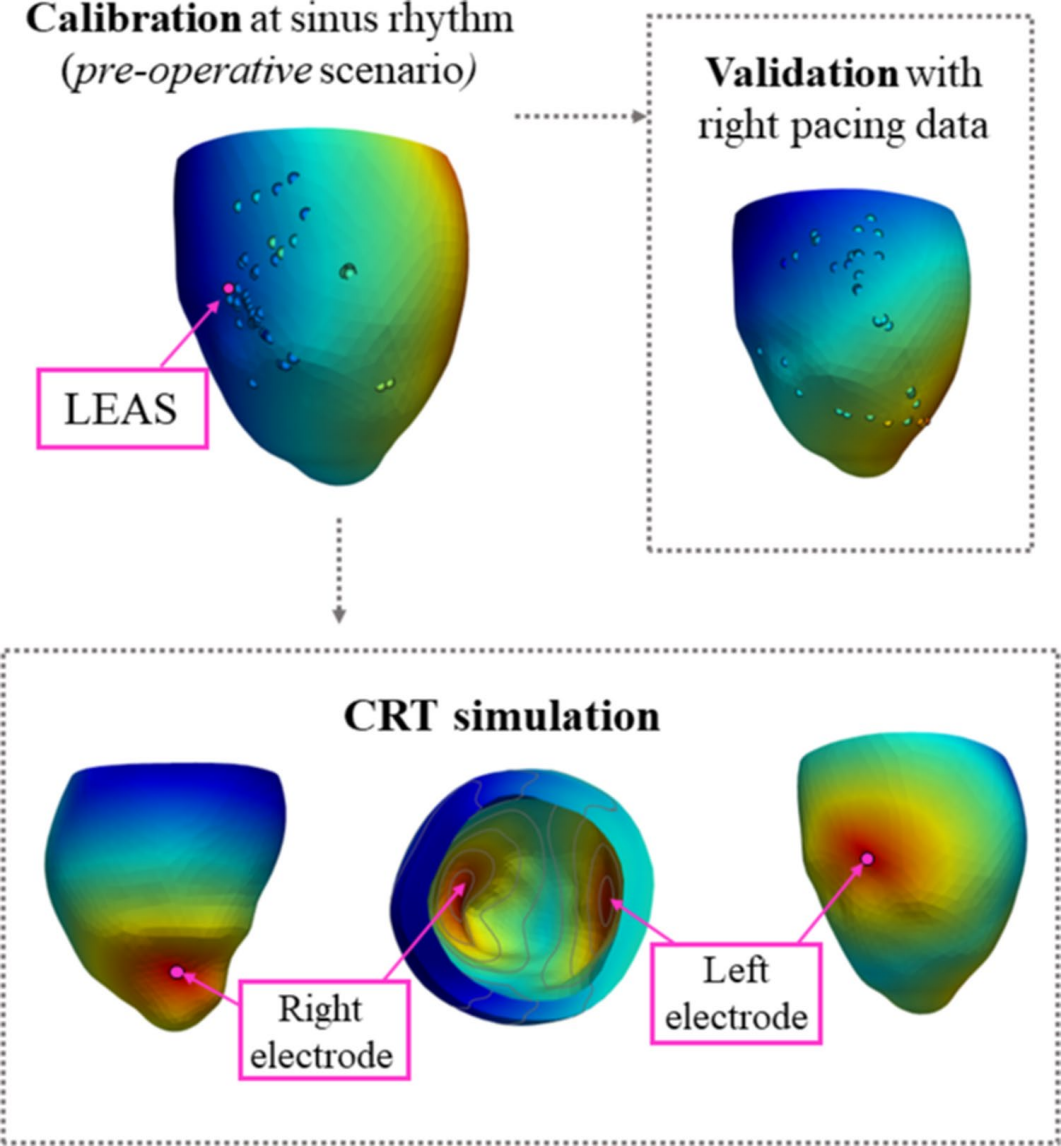
The three scenarios considered in this work: Calibration at sinus rhythm, validation with right pacing, CRT simulations

In this study, we investigate the effect on CRT outcomes induced by three virtual modifications of the implant (see Fig. 4, bottom):

- The left lead position is virtually placed in the LEAS (simulating a common practice of cardiologists in S. Maria del Carmine Hospital) and in other permitted positions along the reconstructed epicardial veins. In this case the right lead is by default located in the LV epicardium (representing the RV apical endocardium) below the interventricular septum and close to the apex (Del Greco et al. 2017);
- The right lead position is also virtually placed in different points along the ventricle septum, keeping the left electrode fixed at LEAS;
- The Ventriculo-Ventricular Delay (VVD) is virtually changed to account for different plausible values used by clinicians, with the convention that positive values indicate that the right stimulation precedes the left one.

We notice that for patients P8 and P11 the fibrotic region spreads out in a wide region, so that a limited (null for P11) area of healthy tissue is covered by the reconstructed epicardial veins. Thus, for these cases the location of the left electrode may be in a fibrotic region, see Results and Discussion sections.

In this work, we decided to use the preload values (i.e. diastolic pressure and volume) resulting from pre-operative measures also in the CRT scenarios. It is known that atrio-ventricular delay induced by the electrode located in the right atrium could however lead to different preload configurations during CRT (Auricchio et al. 2002). This aspect has been here neglected since we are not considering the right atrium pacing.

## Results

### Validation of epicardial veins reconstruction

As highlighted in Sect. 2.2, the reconstruction of epicardial veins, useful for virtually changing the left electrode position, was obtained by means of the electrical activation times acquired by EAMS, as described in Stella et al. (2020). In order to show the validity of such strategy, we present here a comparison for P12 between this reconstruction and the standard one based on CT images at disposal, described in Sect. 2.2.

**Fig. 5:**
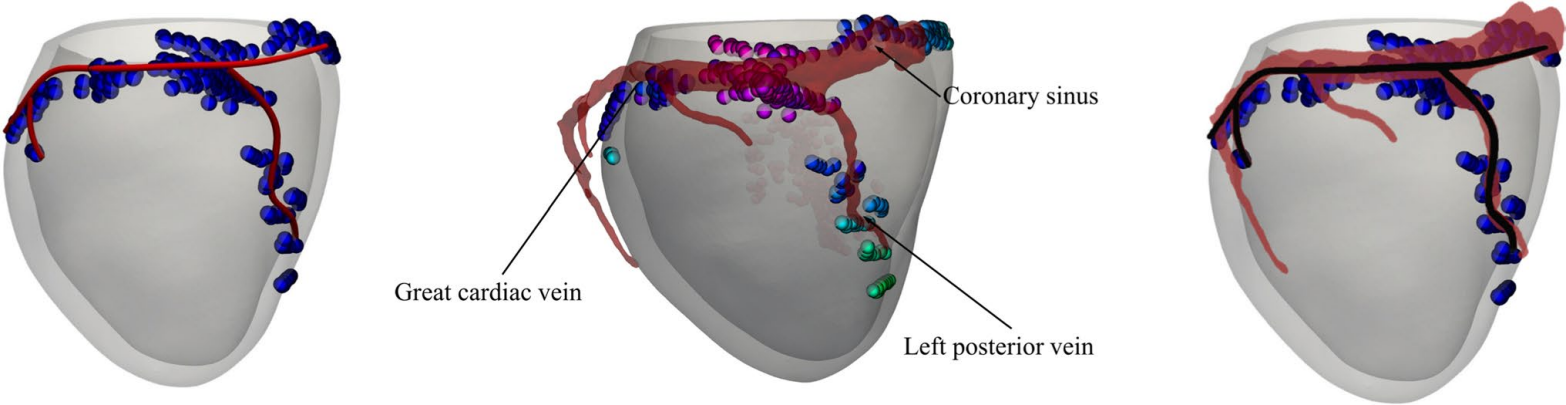
Validation of the reconstructed veins of patient P12 using EAMS measures (on the left) versus CT scan acquired image (in transparency in the middle) and superimposition of the two reconstructions (on the right)

In Fig. 5 we show the results of such comparison. In particular, from the superimposition of the two reconstructions (see subfigure on the right), we found that the CT reconstruction and our EAMS-based reconstruction of the coronary sinus, left posterior vein and great cardiac vein are in excellent agreement, since the latter is completely contained in the former, a part for a small segment located at the end of the left posterior vein. By computing the error between the two reconstructions defined, for each point of the spline obtained by EAMS, as the minimum distance wrt the CT reconstruction centreline, we found that the maximum error is equal to 3.1*mm* and was found precisely at the end of the left posterior vein. This highlights the suitability of the method proposed in Stella et al. (2020) and used in this work to identify the epicardial veins.

### Calibration and validation

**Fig. 6:**
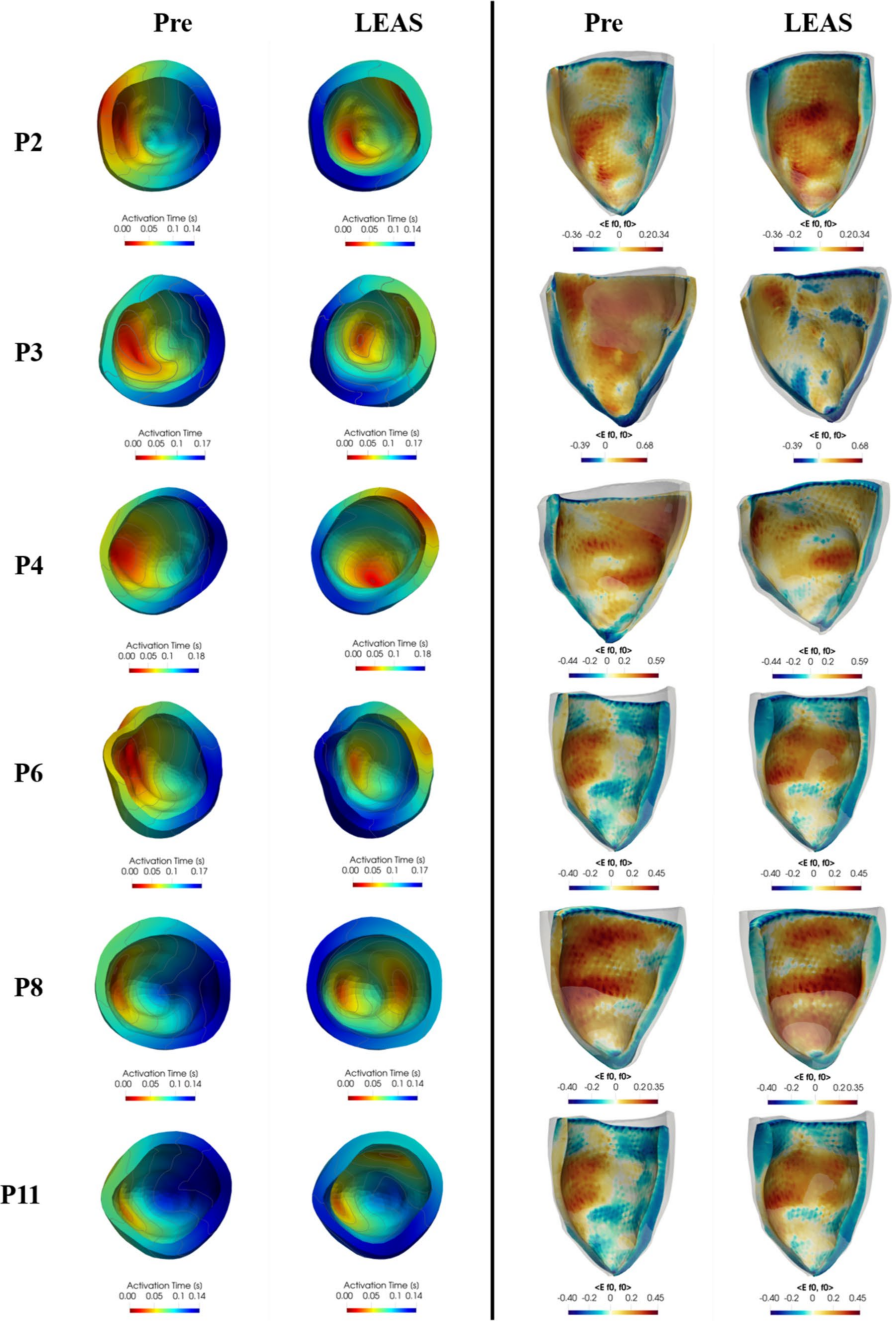
For each patient, comparison at a selected time of electrical activation times plotted in the reference configuration (left part) and fibre direction strain (right part) represented in the deformed configuration, between the pre-operative scenario (left in each of the two parts of the figure) and the LEAS-based CRT virtual scenario (right)

The results of the personalized pre-operative simulations are presented in Fig. 6, first and third columns. We notice that we have two classes of patients, namely the non-fibrotic ones (P2, P3, P4) and the fibrotic ones (P6, P8, P11).

**Table 4:** (i) Personalized electrical, mechanical and Windkessel parameters. (ii) Calibration and validation errors and errors $e_I$ and $e_{II}$ used to assess the validity of our cross-validation (except for P11 because of missing data of right pacing)

	P2	P3	P4	P6	P8	P11	
Electrical							
${\widehat{\sigma }}_f \ [10^{-4} \cdot {\square \text {m}\text {s}^{-1}}]$	2.29	1.99	1.37	1.91	1.91	1.57	Conductivity along the fibres
							normalized wrt the surface-to-volume ratio
							and the transmembrane capacitance
${\widehat{\sigma }}_s \ [10^{-4} \cdot {\square \text {m}\text {s}^{-1}}]$	1.05	0.91	0.62	0.87	0.87	0.72	Normalized conductivity—sheets direction
${\widehat{\sigma }}_n \ [10^{-4} \cdot {\square \text {m}\text {s}^{-1}}]$	0.34	0.29	0.20	0.28	0.28	0.23	Normalized conductivity—normal direction
$c_0 \ [{\text {s}^{-\frac{1}{2}}}]$	84.37	73.36	77.03	73.36	80.70	73.36	Velocity of the depolarization
							wave along the fibre direction
							for a planar wavefront
$\varepsilon \ [-]$	11.96	14.95	14.20	19.46	14.95	14.95	Impact of the wavefront
							curvature on the propagation velocity
$z \in [0, 1] \ [-]$	1.0	1.0	1.0	0.7	0.7	0.9	Decreased electrical conductivity
							and active contractility in fibrotic regions
							($z = 1$ denotes the healthy tissue);
							Tissue stiffening and reduced contractility
							by a stiffness rescaling of $[z + (1 - z)4.56]$
*Calibration error* $[\%]$	4.0	6.1	7.3	5.9	9.9	4.6	
*Validation error* $[\%]$	7.8	4.9	4.1	3.4	6.0	–	
$e_I$ $[\%]$	14.4	18.8	15.8	44.1	6.5	–	
$e_{II}$ $[\%]$	12.9	13.7	25.4	49.2	12.7	–	
Mechanical							
$a_{\text {XB}} \ [10^2 \cdot {\text {M}\text {Pa}}]$	2.70	3.07	3.21	2.66	1.87	2.06	Myocardial tissue contractility
Windkessel							
$R \ [10^{7} \cdot {\text {Pa} \text {s} \text {m}^-3}]$	3.40	3.00	3.92	2.8	5.60	3.50	Resistance of the two-element
							Windkessel model for the circulatory
							system during the ejection phase

In Table 4 we report the parameters values obtained from the calibration of the electrical, mechanical and 0D hemodynamics models, as well as the calibration errors obtained for the electrical function and computed using (2), measuring the discrepancy between the EM pre-operative simulation and the pre-operative clinical data acquired at the coronary sinus (see Table 2b). It is worth noticing that they are all below the $10 \%$ threshold. In particular, calibration of patient P8 led to the highest error, probably due to the smaller number of available measures of activation times registered at the coronary sinus with respect to the other patients (see Table 2b). Notice that the mechanical calibration errors are identically zero and thus they are not reported. This is due to the global nature of such measures (volumes and pressures).

In order to assess the suitability of the calibration of the electrical parameters obtained by means of pre-operative activation times, we performed a validation by comparing the numerical results of the Eikonal problem against data not used in the calibration itself (cross-validation). To this aim, we used the activation times acquired with EAMS during right pacing procedure we had at disposal (only right electrode at the apex is stimulating, see "Right Pacing" column of Table 2b) and we performed numerical simulations in the right pacing scenario (see Fig. 4, top). In Table 4 we report the values of the relative errors obtained by this validation procedure (validation error), using a formula analogous to (2). We observe the excellent matching between the computed and the measured activation times in right pacing conditions.

As shown in Table 2b, the number of acquisition points in the two conditions (sinus rhythm and right pacing) is different, as well as the measured activation time ranges. To prove the effectiveness of our validation procedure we quantitatively measure the "distance" between the two sets of data. Actually, since the data sets are not directly comparable by means of correlation matrices or coefficients because of their different dimensions, we computed the error $e_I$ between the activation times measured during right pacing conditions and the activation times computed by our simulation during sinus rhythm, at the points where the former set of data is available. If such errors are large (in comparison with validation errors), thus we can infer that the two set of data are sufficiently different: indeed, if they were not, $e_I$ would be small since the simulation personalized at sinus rhythm would be able to fit well the right pacing data. In a dual way, we computed the error $e_{II}$ between the activation times measured at sinus rhythm conditions and the activation times computed by our simulation during right pacing, at the points where the former set of data is available. From the results reported in Table 4, we can see that such errors are large in comparison with validation errors. We can definitively state that our calibration has been successfully cross-validated.

We notice that we did not have at disposal pressures and volumes for the right pacing condition, so that it was not possible to perform a mechanical validation.

The personalized parameters are then used in turn for the simulations of the virtual CRT scenarios, whose analysis is presented in the next paragraphs.

In Fig. 6 we report for all the patients the activation times and the fibre direction strain $<E\varvec{f}_0,\varvec{f}_0>$ in the deformed configuration (together with the reference configuration in background) at a representative time instant for both the pre-operative and the LEAS-CRT scenarios. Notice that this quantity has been computed wrt the reference configuration, in accordance with its rigorous definition (Gurtin et al. 2010) and with other works in the same field, see, e.g. Strocchi et al. (2020). Moreover, the results of fibre direction strain and of the displacements provide an evidence that LEAS-CRT improves synchrony with respect to the pre-operative case. This could be highlighted both by the displacements (difference between the coloured and the background/grey/reference geometries), which are more asymmetric in the Pre case, and by the strain plots, which are in general less uniform in the Pre case.

Notice that the activation times of the fibrotic cases P6,P8,P11 are in accordance with the presence of fibrosis depicted in Fig. 2. Also, we notice a less homogeneous strain distribution for such cases, especially for P6 and P11.

### Optimal left lead location

**Fig. 7:**
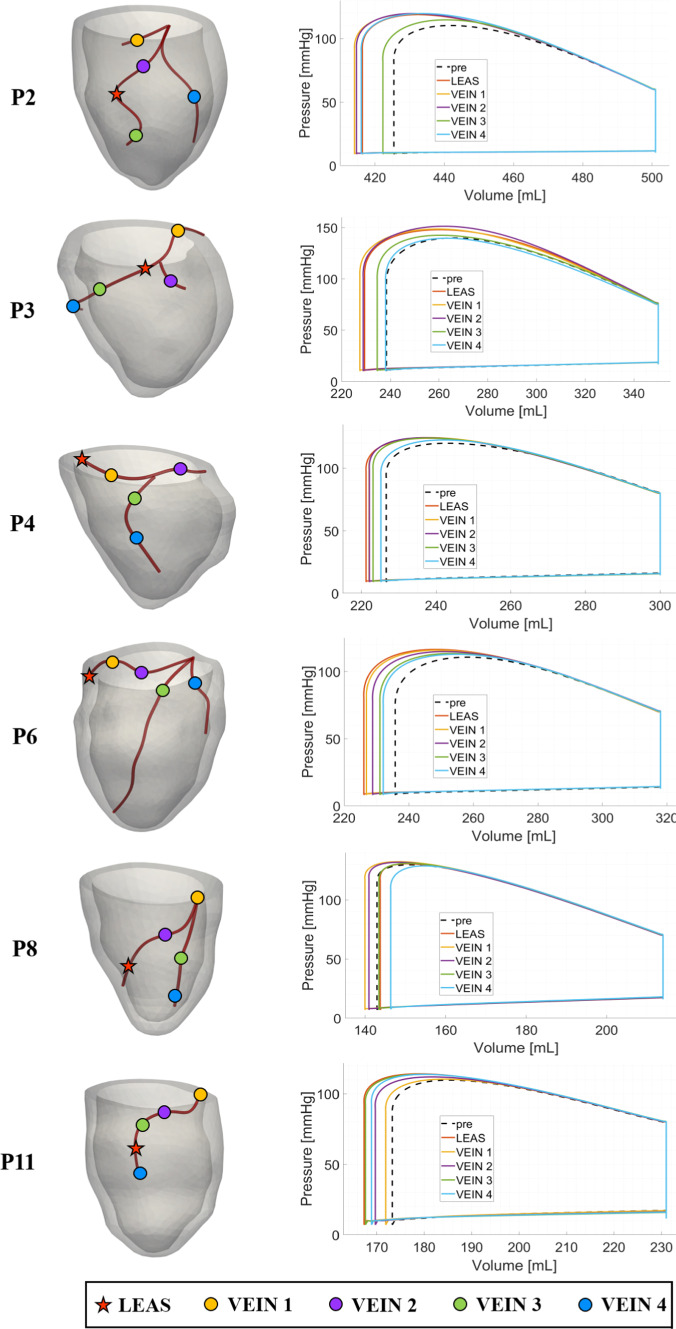
Representative choices of left electrode locations for virtual CRT simulations (left) and corresponding PV loops (right), overlapped to the pre-operative scenario

In Fig. 7, left, we report our choices about the anatomically compatible locations inside the epicardial veins of the left electrode considered in our virtual CRT simulations.

**Table 5:** Biomarkers corresponding to virtual CRT configurations varying left lead position. See Fig. 7 for the different locations of the left electrode. For CRT scenarios we indicate the percentage of variation of $dP/dt|_{\text {max}}$, *SV* and *SW* with respect to the pre-operative case, whereas for *EF* we indicate the absolute changes. P2, P3, P4: non-fibrotic cases; P6, P8, P11: fibrotic cases

Patient	Location	${dP/dt|}_{{ {max}}}$	${{SV}}$	${{EF}}$	${{SW}}$
		$[{\text {mmHg}\text {s}^{-1}}]$	$[{ml}]$	$[\%]$	$[{\text {mmHg}\,ml}]$
P2	Pre-operative	2252	76	15.1	6300
	*LEAS*	$+7.4\%$	$+11.8\%$	$+1.8$	$+19.4 \%$
	*VEIN 1*	$+8.1\%$	$+14.5\%$	$+2.3$	$+23.5\%$
	*VEIN 4*	$+12.0\%$	$+11.8\%$	$+1.9$	$+20.1\%$
P3	Pre-operative	3119	112	31.9	11, 600
	*LEAS*	$+13.0\%$	$+8.0\%$	$+2.6$	$+14.8\%$
	*VEIN 1*	$+9.7\%$	$+9.8\%$	$+3.1$	$+16.5\%$
	*VEIN 2*	$+13.9\%$	$+8.0\%$	$+2.7$	$+17.3\%$
P4	Pre-operative	2278	73	24.5	6800
	*LEAS*	$+6.5\%$	$+7.3\%$	$+1.8$	$+10.8\%$
	*VEIN 1*	$+3.1\%$	$+6.3\%$	$+1.5$	$+10.0\%$
	*VEIN 4*	$+6.6\%$	$+1.9\%$	$+0.5$	$+3.7\%$
P6	Pre-operative	2186	82	25.9	7000
	*LEAS*	$+3.7\%$	$+11.8\%$	$+3.0$	$+16.9\%$
	*VEIN 1*	$+2.0\%$	$+10.8\%$	$+2.8$	$+15.4\%$
P8	Pre-operative	2731	70	32.9	6700
	*LEAS*	$-7.5\%$	$-1.4\%$	$-0.3$	$-1.6\%$
	*VEIN 1*	$+2.0\%$	$+4.2\%$	$+1.4$	$+6.6\%$
P11	Pre-operative	2460	58	25.0	4900
	*LEAS*	$+0.7\%$	$+10.3\%$	$+2.6$	$+15.6\%$
	*VEIN 4*	$+1.5\%$	$+6.9\%$	$+1.9$	$+12.4\%$

In the same figure, on the right we report and compare the corresponding PV loops. Notice that for all the patients the Stroke Volume (SV) (i.e. difference between diastolic and systolic volumes, $EDV - ESV$), and thus the Ejection Fraction $EF = SV/EDV$, increase for almost all virtual CRT scenarios with respect to the pre-operative case.

In Table 5 we report the specific values of *SV* and *EF* for the most representative virtual CRT scenarios (see Table 8 in the Appendix for the complete list of values).

From these results, we first notice that for the non-fibrotic cases LEAS is an effective choice in terms of improvements of the two biomarkers with respect to the pre-operative cases. Moreover, we notice that for P2 and P3 it is possible to obtain a further improvement by selecting a different left electrode location. Also for the fibrotic cases significant improvements of *SV* and *EF* were found for P6 and P11, with no further improvement by changing the left electrode location. Instead, for P8 LEAS reveals to be a non-optimal choice, with VEIN1 location which is able to provide a small improvement. Summarizing we have a maximum improvement with respect to the pre-operative case of *SV* in the percentage range $[7.3 \%, 14.5 \%]$ for the non-fibrotic cases and of $[4.2 \%, 11.8 \%]$ for the fibrotic cases; for *EF* we have a maximum improvement in the absolute range [1.4, 3.1] (no significant differences between the two groups).

In Table 5 other biomarkers are presented. Specifically, we computed the maximal LV pressure gradient $dP/dt|_{\text {max}}$ (Thibault et al. 2012) occurring during systole. $dP/dt|_{\text {max}}$ is used as an index of ventricular performance ( Zweerink et al. (2019); Kass et al. (1987)) and thus elevated values of $dP/dt|_{\text {max}}$ may be a sign, during the acute phase, of significant contractility and resynchronization (Spragg et al. 2010). Again, we notice that LEAS is an effective choice for the non-fibrotic cases, whereas for fibrotic case no significant improvements are noticed. Moreover, for P2 and P3 different left electrode locations provide a further improvement in terms of $dP/dt|_{\text {max}}$. In any case, we notice that for all the CRT scenarios this index features larger values than the pre-operative case, being maximum improvements with respect to pre-operative case in the percentage range $[6.6 \%, 13.9 \%]$ for the non-fibrotic cases and $[1.5 \%, 3.7 \%]$ for the fibrotic cases. Another useful index reported in Table 5 is the Stroke Work (*SW*) defined as the work done by the ventricle to eject the blood and corresponding to the area within the PV loop. Analogously to the other biomarkers, LEAS is shown to be in general an effective choice and the value resulting from CRT simulations significantly increases with respect to pre-operative case in the ranges $[10.8 \%, 23.5\%]$ for the non-fibrotic cases and $[6.6 \%, 16.9\%]$ for the fibrotic cases.

### Study of the ventriculo-ventricular delay

**Fig. 8:**
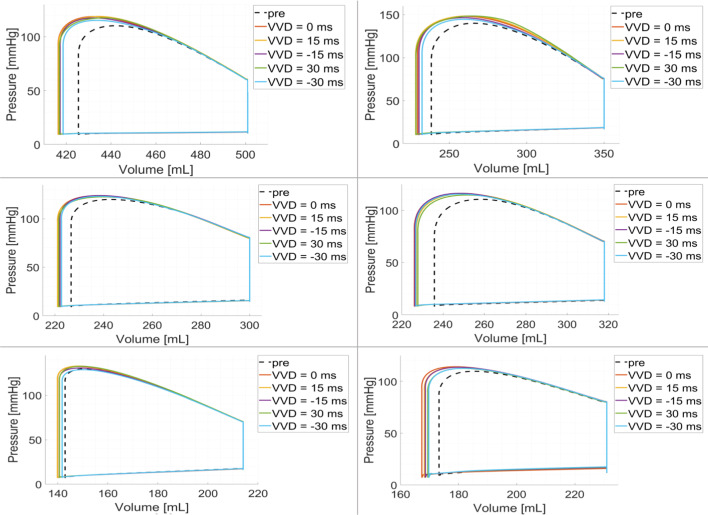
PV loops of the virtual CRT simulations at LEAS for five different choices of ventriculo-ventricular delay (*VVD*), overlapped to the pre-operative scenario

In Fig. 8 we report the PV loops obtained by using LEAS for the left electrode and varying the ventriculo-ventricular delay with the values $VVD = [-30, \ -15, \ 15, \ 30] \ ms$, with the convention that positive values indicate that the right stimulus anticipates the left one.

**Table 6:** Biomarkers corresponding to virtual CRT configurations varying Ventriculo-Ventricular Delay (*VVD*) with the left electrode positioned at LEAS. Positive values of *VVD* mean that the right stimulation occurs before the left one. For CRT scenarios we indicate the percentage of variation of $dP/dt|_{\text {max}}$, *SV* and *SW* with respect to the pre-operative case, whereas for *EF* we indicate the absolute changes. P2, P3, P4: non-fibrotic cases; P6, P8, P11: fibrotic cases

Patient	Delay	${dP/dt|}_{{ {max}}}$	${ {SV}}$	${ {EF}}$	${ {SW}}$
	[ms]	$[{\text {mmHg}\text {s}^{-1}}]$	$[{ml}]$	$[\%]$	$[{\text {mmHg}\,ml}]$
P2	*Pre-operative*	2, 252	76	15.1	6, 300
	$-15$	$+5.2\%$	$+10.5\%$	$+1.6$	$+17.0\%$
	0	$+7.4\%$	$+11.8\%$	$+1.8$	$+19.0\%$
	30	$+11.3\%$	$+10.5\%$	$+1.7$	$+18.0\%$
P3	*Pre-operative*	3, 119	112	31.9	11, 600
	$-15$	$+7.0\%$	$+7.1\%$	$+2.3$	$+12.2\%$
	0	$+13.0\%$	$+8.0\%$	$+2.6$	$+14.8\%$
	30	$+24.9\%$	$+8.9\%$	$+2.8$	$+16.6\%$
P4	*Pre-operative*	2, 278	73	24.5	6, 800
	$-15$	$+1.2\%$	$+6.2\%$	$+1.5$	$+9.8\%$
	0	$+6.5\%$	$+7.3\%$	$+1.8$	$+10.8\%$
	15	$+9.8\%$	$+7.6\%$	$+1.8$	$+10.9\%$
P6	*Pre-operative*	2, 186	82	25.9	7, 000
	$-15$	$+2.4\%$	$+11.5\%$	$+3.0$	$+16.6\%$
	0	$+3.7\%$	$+11.8\%$	$+3.0$	$+16.9\%$
	30	$+6.7\%$	$+9.7\%$	$+2.5$	$+13.4\%$
P8	*Pre-operative*	2, 731	70	32.9	6, 700
	$-15$	$-0.9\%$	$+2.8\%$	$+1.1$	$+6.9\%$
	0	$+2.0\%$	$4.2\%$	$+1.4$	$+6.6\%$
	15	$+3.0\%$	$+4.2\%$	$+1.5$	$+6.9\%$
P11	*Pre-operative*	2, 460	58	25.0	4, 900
	$-15$	$+0.5\%$	$+6.9\%$	$+2.1$	$+12.4\%$
	0	$+0.7\%$	$+10.3\%$	$+2.6$	$+15.6\%$
	15	$-0.9\%$	$+8.6\%$	$+2.2$	$+13.2\%$

We observe that we obtain quite similar behaviours to the $VVD = 0$ scenario, in any case with an increased stroke volume with respect to the pre-operative one. Negative *VVD*, specifically $VVD = -30$ ms, seems to provide however slightly worse improvements.

These observations are confirmed in Table 6, where we report the same biomarkers of the previous analysis for the best positive and negative values of *VVD* (see Table 9 in the Appendix for the complete list of values). In particular, for the non-fibrotic cases we observe that positive values of *VVD* always allow to improve the four biomarkers with respect to the *synchronous* case $VVD = 0$, especially for $dP/dt|_{\text {max}}$. Instead, negative values of *VVD*, although improving the biomarkers with respect to the pre-operative case, do not seem to perform better than the synchronous case. As regards the fibrotic cases, we observe not significant improvements of the delayed cases with respect to the synchronous case.

### Study of the right electrode location

**Fig. 9:**
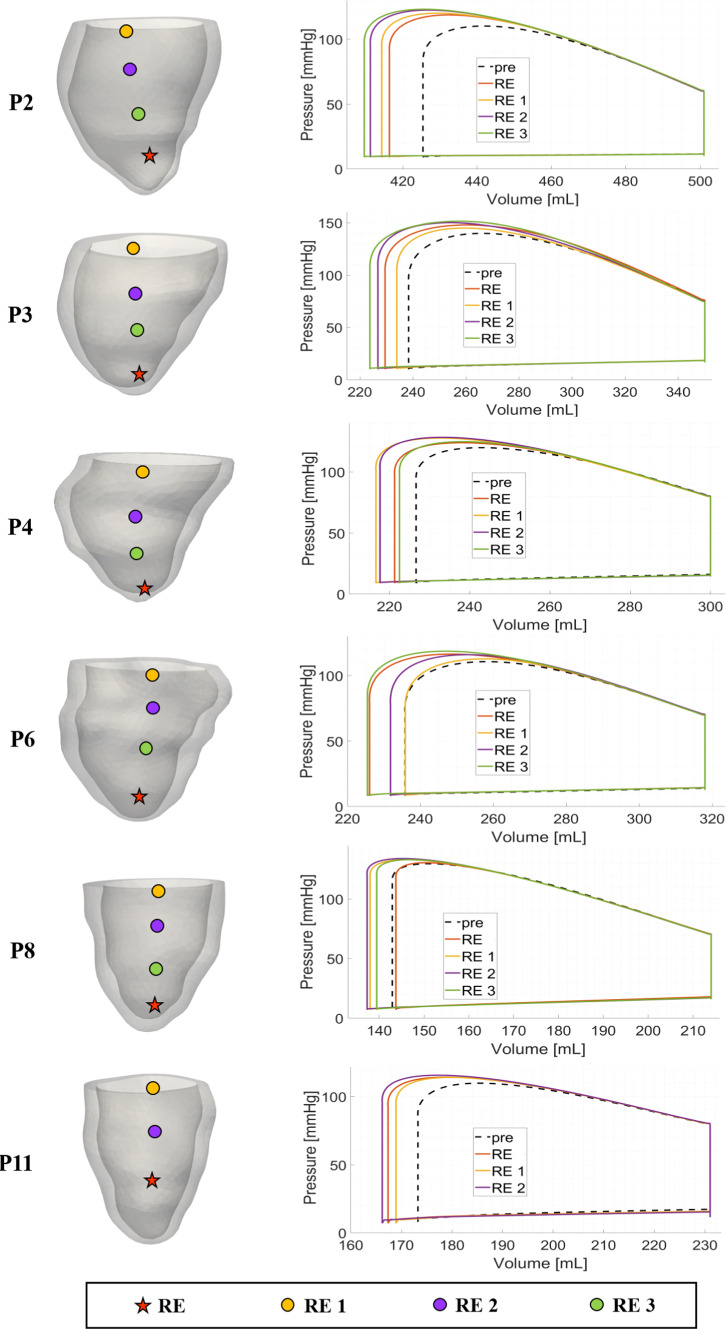
Choices of Right Electrode locations (RE) for virtual CRT simulations (left) and corresponding PV loops (right), overlapped to the pre-operative scenario. Left electrode placed at LEAS and no *VVD* for all the cases

In this section, we report the results obtained by varying the position of the right electrode along the interventricular septum, while keeping the left one fixed at LEAS and without any *VVD*. In Fig. 9 we can observe the choices of right electrode locations for each patient, together with the corresponding PV loops. From these results we notice that there is a significant improvement of the stroke volume when the right electrode is located in the septal region halfway between the apex and the base. More in detail, from Table 7, where we report the best cases together with the standard one, we observe that for the non-fibrotic cases the improvements with respect to the pre-operative case in terms of *SV*, *EF* and *SW* almost double compared to the standard apical *RE* case (see Table 10 in the Appendix for the complete list of results). As regards the fibrotic cases, there is still an improvement of the three biomarkers compared to the *RE* case, even if milder.

On the other hand, for the non-fibrotic cases, $dP/dt|_{\text {max}}$ does not appear to benefit from the upward movement of the right electrode along the septum, whereas slight improvements are noticed for the fibrotic cases.

**Table 7:** Biomarkers corresponding to *virtual* CRT configurations varying right lead position, with LEAS choice for the left one and $VVD = 0$. *RE* position indicated the apical position used in the numerical experiments reported in Sects. 3.3 and 3.4. See Fig. 9 for the different locations of the right electrode. For CRT scenarios we indicate the percentage of variation of $dP/dt|_{\text {max}}$, *SV* and *SW* with respect to the pre-operative case, whereas for *EF* we indicate the absolute changes. P2, P3, P4: non-fibrotic cases; P6, P8, P11: fibrotic cases

Patient	Location	${dP/dt|}_{{{max}}}$	${ {SV}}$	${{EF}}$	${ {SW}}$
		$[{\text {mmHg}\text {s}^{-1}}]$	$[{ml}]$	$[\%]$	$[{\text {mmHg}\,ml}]$
P2	Pre-operative	2252	76	15.1	6300
	*RE*	$+7.4\%$	$+11.8\%$	$+1.8$	$+19.4 \%$
	*RE 3*	$+3.5\%$	$+19.7\%$	$+3.1$	$+33.4\%$
P3	Pre-operative	3, 119	112	31.9	11, 600
	*RE*	$+13.0\%$	$+8.0\%$	$+2.6$	$+14.8 \%$
	*RE 3*	$+14.5\%$	$+12.5\%$	$+4.2$	$+22.3\%$
P4	Pre-operative	2278	73	24.5	6800
	*RE*	$+6.5\%$	$+7.3\%$	$+1.8$	$+10.8\%$
	*RE 2*	$+7.9\%$	$+12.2\%$	$+3.0$	$+19.3\%$
P6	Pre-operative	2186	82	25.9	7000
	*RE*	$+3.7\%$	$+11.8\%$	$+3.0$	$+16.9\%$
	*RE 3*	$+7.9\%$	$+12.5\%$	$+3.2$	$+19.3\%$
P8	Pre-operative	2731	71	33.2	6700
	*RE*	$-7.5\%$	$-1.4\%$	$-0.3$	$-1.6\%$
	*RE 2*	$-5.1\%$	$+8.5\%$	$+2.6$	$+11.3\%$
P11	Pre-operative	2460	58	25.0	4900
	*RE*	$+0.7\%$	$+10.3\%$	$+2.6$	$+15.6\%$
	*RE 2*	$+2.6\%$	$+12.1\%$	$+3.1$	$+19.6\%$

## Discussion

In this work we performed a computational study to assess the performance of CRT in terms of relevant biomarkers in different scenarios obtained by virtually varying the electrodes locations and their phase shift. To do this we carried out EM numerical simulations in three non-fibrotic and three fibrotic patients, suitably personalized by means of EAMS and mechanical data (acquired during the standard CRT procedure, with no additional invasive procedures), and compared some hemodynamic outputs of clinical interest resulting from the 0D circulation model coupled with the EM model. Our computational model incorporates an ionic model and a force generation model that are able to faithfully capture the frequency-dependent effects on the active force generation process, resulting in a greater contractility as heart rate increases, thus accounting for the different heart rates characterizing each patient.

This work featured some novelties in the framework of computational CRT studies:

- The validation of the electrophysiology part of the personalized EM model against EAMS measures obtained in a scenario (right pacing) different from that used for the calibration (sinus rhythm);
- The use of the personalized EM model in the context of CRT optimization;
- The use of the reconstruction of epicardial veins to identify anatomically compatible locations of the left electrode;
- The analysis of the CRT performance when different locations of the right electrode are exploited.

We studied cases both with and without fibrotic regions. For the first cases, we have used the bullseye diagram available for the patient from LGE-MRI. Specifically, for most clinical indications, the presence of myocardial fibrosis is defined as an area of high signal intensity as bright as the LV blood pool can be evaluated qualitatively, and reported using the 17-segment (bullseye) model recommended by the AHA. In our clinical practice we do not have a software for quantitative analysis of fibrosis, but we do a qualitative evaluation of the post-contrast images.

The results of the EM numerical simulations were evaluated by computing four clinically relevant biomarkers just after the virtual CRT implantation, i.e. in acute conditions. To obtain them, we used the same personalized parameters of the pre-operative case, due to the fact that the cardiac muscle features changes of electrical and mechanical properties after over 6 months, as pointed out in Sect. 2.6. The Stroke Volume (*SV*) and the Ejection Fraction (*EF*) were used as preliminary and easily computable indices of the restored synchronicity (for computational studies see, e.g. Isotani et al. (2020)), although it is known that they are more reliable indicators when computed after chronic reverse remodelling following CRT (Abraham et al. 2002; Sutton et al. 2003; Steendijk et al. 2006) rather than measured acutely, i.e. just after the procedure. As a further index, we proposed to use the Stroke Work (*SW*) that accounts also for the pressure jump within the cardiac cycle. In addition to *EF* and *SV*, that monitor the amount of blood ejected, *SW* quantifies the work performed by the heart to pump that amount of blood into the circulatory system. The joint evaluation of the mentioned indices allows for a better assessment of the cardiac function. We also analysed the maximum temporal gradient of pressure during a cardiac cycle, $dP/dt|_{max}$, employed to evaluate the synchronization and contractility of the cardiac muscle. This index has been used in other computational studies, e.g. in Sermesant et al. (2012); Pluijmert et al. (2016); Isotani et al. (2020); Fan et al. (2022); Desrues (2023), and it has been shown to be significant also in the acute regime (Singh et al. 2006; Spragg et al. 2010), since its acute increase of about 10% or more predicts reverse remodelling (Duckett et al. 2011). Future studies should include in the mathematical description also a remodelling model based for example on the coupling with a complete 0D network of the systemic circulation and on feedback of this on the cardiac function to adjust resistances and contractility to the new post-operative situation. This will require an important effort from the theoretical point of view, even if some researchers have already started to work in this direction, see e.g. Lee et al. (2016).

As regards the positioning of the left electrode, the clinical practice considers epicardial veins as a natural way to place it, for example in a lateral or posterolateral tributary of the coronary sinus (Breithardt et al. 2003; Vaillant et al. 2013; Wang et al. 2022) or in anterolateral and posterolateral locations (Dong et al. 2012). Some studies proposed to consider measures of activation, for example coming from EAMS, and to locate the left electrode in the site with latest electrical activation (LEAS) as commonly performed at S. Maria del Carmine Hospital (Singh et al. 2006; Del Greco et al. 2017). Other studies highlighted that the best location is specific to each individual, so it does not always match the latest activated segment (Derval et al. 2010). Our results showed that the LEAS is an effective choice to position the left electrode for the non-fibrotic cases, in terms of improvements of the biomarkers with respect to the pre-operative case. This is in accordance with previous computational studies, see, e.g. Albatat et al. (2021). Notice that our LEAS always falls in lateral (P6, P8), antero-lateral (P2, P4), or in anterior (P11) position. Moreover, we showed how it is possible to identify other locations in the epicardial veins that could even slightly improve the performance of LEAS. A validation of the optimality of the left electrode location should be in future performed for example by comparing the values of $dP/dt|_{max}$ with patient’s data. However, this requires invasive measures which are not the standard practice during CRT procedures. This will be possible owing to *ad hoc* research studies beyond the clinical practice.

As regards the ventriculo-ventricular delay, our results showed that for the non-fibrotic cases the activation of the right electrode before (15 or $30 \ {\text {m}\text {s}}$) the left one seems to improve the CRT performance. This is in contrast with the clinical experience, where the left electrode is usually activated first, to compensate the delay of activation of the corresponding region (Dreger et al. 2012), and with some computational studies, see, e.g. Villongco et al. (2016). However, some work has contradicted this indication, showing that there is no effective agreed rule in this regard (Porciani et al. 2005; Boriani et al. 2009). The interpretation of our findings relies on the observation that in the physiological case the region where the right electrode is usually placed is activated by the Purkinje network slightly earlier than the region where the left electrode is located. Thus, in healthy conditions the front should propagate from the interventricular septum through the bundle of His and subsequently reach the left lateral portion of the LV by means of the Purkinje network. Thus, during CRT, anticipating the right stimulus could restore a more physiological condition in terms of activation, in comparison to the scenario when the left electrode is activated first. Notice that a restored physiological activation sequence does not necessarily imply an improved synchrony of the ventricular contraction and rotation (Gerach et al. 2021).

One of the novelties of this work relies on exploring different possible locations of the right electrode along the septum. In the clinical practice, usually this is placed in the apical position. However, some clinical studies suggested that other locations could be explored, see for example Vaillant et al. (2013); Worsnick et al. (2018); Wang et al. (2022), where the authors proposed to place the right electrode in the mid-septum or in outflow tract septum. Accordingly, we virtually moved it in other positions which are compatible with anchoring by screwing to the septum (Worsnick et al. 2018), to understand if the performance of CRT could improve. We found that in general the performance in terms of *EF*, *SV* and *SW* seems to improve by positioning the electrode a little higher, towards the base. Although very preliminary and far to provide specific indications, we believe that this new analysis could provide a new horizon of exploration for the improvement of CRT and deserves further investigations.

One of the critical issues related to the good functioning of the therapy is the presence of fibrosis. Some clinical studies revealed that the presence of fibrosis (e.g. midwall fibrosis) could be a predictor of poor response to CRT. This is because the more the muscle is replaced by fibrosis, the more difficult it will be to recover its contractile function (Ypenburg et al. 2007; Leyva et al. 2012; Massoullié et al. 2019). For example in Ypenburg et al. (2007) the authors showed that the non-responders mainly consist of patients with scar tissue in the region of the left lead or with wide scar tissue. Our results went in this direction, highlighting that the improvements of all biomarkers are in general less noticeable than in the non-fibrotic cases. See also Costa et al. (2019), where the authors computationally found that pacing at the LV epicardial surface in proximity to scar increases the volume of high repolarization gradients, thus explaining the CRT-induced ventricular tachycardia in patients with ischemia. Again, further investigations will be mandatory, for example to find possible correlations between the fibrosis region and the placement of the electrodes.

We would like to acknowledge that in the last years Conduction System Pacing (CSP) has become a valid alternative to CRT. This consists in pacing directly the ventricular conduction system and it has the potential to provide physiological-paced activation (Catanzariti et al. 2013). CRT remains recommended for patients with HF, LVEF less than 35%, LBBB with QRS duration > 130 ms or no LBBB morphology with QRS duration > 150 ms, and New York Heart Association class II-IV symptoms. New recommendations are made for CSP when CRT cannot be performed. Nowadays, data on CSP are mainly observational; ongoing studies are investigating future indications on the use of CSP compared to CRT (Chung et al. 2023). In this direction, recent computational studies investigated the CSP performance applying different pacing strategies and highlighted differences with respect to CRT (Meiburg et al. 2023; Strocchi et al. 2023).

Finally, we remark that all the results of this work were found under a main assumption: the personalized *pre-operative* parameters are considered to remain the same just after the CRT. This means that the electrical and mechanical properties of the heart muscle and the blood resistances do not change immediately after the implantation, since the LV needs over six months to experience remodelling (Sutton et al. 2003). This allowed us to use the pre-operative personalized electro-mechanical parameters also for the virtual CRT scenarios.

To conclude, in this paper, we have showcased the practical application of our developed methodology on some specific patients. Our intention was not to conduct extensive statistical analyses, given the relatively modest sample size. Instead, our focus was on presenting a proof-of-concept to highlight the potential of the proposed methodology. The proposed pipeline was particularly well-suited for systematic replication on a patient-specific basis. This methodology could serve as the foundation for a routine procedure aimed at optimizing CRT with consideration for individualized factors, including geometric, electrophysiological, and mechanical aspects, and their complex interplay.

### Limitations and further developments

There are several limitations in this work, which are discussed in what follows.

- We did not consider in our simulations the left atrium. This means that we were not able to take into account possible preload changes due to different atrio-ventricular delays (Hu et al. 2013). Including this effect might change the optimal position of the leads. Thus, our results should be understood as obtained under the hypothesis of sinus-rhythm atrio-ventricular delay;
- Our electrical calibration was based on measures obtained by means of an Electro-Anatomical Mapping System. Not all CRT interventions are nowadays made exploiting this procedure to guide the insertion of the electrodes and limit the radiation exposure. However, in the last years performing EAMS-assisted CRT is becoming more and more frequent, as proved by the multicentric study (Del Greco et al. 2017);
- We did not consider the Purkinje system in our electro-mechanical model. This simplification could have an impact on the results, due, e.g. to possible retrograde propagation through the Purkinje network originating from the CRT pacing electrodes (Landajuela et al. 2018; Strocchi et al. 2023). However, at the heart rates simulated in this study, re-entries are very unlikely to form making this limitation more relevant in studies of arrhythmia dynamics;
- Our pipeline, from MRI images and EAMS measures to the numerical results of the virtual CRT scenarios, passing through the electro-mechanical calibration, is nowadays not completely automatic. Some steps are still manual (ventricle segmentation, electrical calibration), whereas other are completely automatic (mechanical calibration, building of nested meshes, generation of epicardial veins). In particular, the computational time for the EM simulations (about 2 h) is compatible with the timings of the EAMS + CRT procedure, whereas the reconstruction (about 5 h) and calibration (about 10 EM simulations) steps are not. In the future, we plan to make our procedure completely automatic and to improve the registration and calibration steps by using a non-manual procedure, allowing to have a total computational time (geometry reconstruction + calibration + CRT scenarios simulations) compatible with the CRT timings, so in principle one could insert it as a module of EAMS.
- Our model calibration and numerical experiments ignore the right ventricle. This is an important limitation of the work since during LBBB the contraction of the right ventricle changes the stretch of the left ventricle free wall, altering its contraction. Second, the pressure of the right ventricle acts on the septum, changing its motion and the strains. This will have major effects, especially when the location of the right ventricular electrode is changed. Thus, this study should be considered mainly as the presentation of some significant novelties for computational studies of CRT (generation of epicardial veins to locate the left stimulus, calibration with patient-specific electrical and mechanical measures, validation against other measures) rather than as a work which provides definitive quantitative results on this topic;
- We also ignored the presence of the right atrium. CRT is often performed by pacing the right atrial appendage together with RV and LV (Gorcsan and Faddis 2020). This should produce probably a minimal effect on the results compared to the exclusion of the right ventricle (see previous point). However, this an important point that deserves future investigations suggesting a whole-heart model as a possible extension;
- We assumed that the geometry acquired through MRI is stress-free. A more fine-tuned procedure would involve estimating the reference configuration by solving the inverse elasticity problem (Govindjee and Mihalic 1996; Marx et al. 2022; Barnafi et al. 2024);
- The present model neglects mechano-electrical feedbacks, which can be related to stretch-activated currents, conductivity tensor strain, and calcium and troponin binding during activation. These phenomena may impact depolarization, repolarization, and spatial pattern heterogeneity (Gerach and Loewe 2024; Salvador et al. 2022);
- We have considered a relatively simplified model of blood circulation, namely a two-element Windkessel. More elaborated models, such as closed-loop full body circulation models, can be considered, see e.g. Gerach et al. (2021); Augustin et al. (2021). However, we deem that the choice of using a simple model fits more in the case where few clinical quantities are available, in which the calibration of more complex models would be severely under-determined and poorly reproducible;
- Our validation procedure relied on right pacing measures. A more complete validation protocol for future studies involving CRT simulations should include also a comparison against measures obtained after the CRT implantation.
- We validate against clinical measures only the electrophysiology part of our personalized EM model. The availability of mechanical measures (displacements, strains, etc.), not used in the mechanical calibration, to perform a validation also of the mechanical part will be fundamental for future studies. Regarding this work, we can then interpret our mechanical results not to be directly clinically relevant, rather to provide preliminary results waiting for validation.
- We finally notice that our results cannot nowadays be reproduced. Indeed, regarding the Finite Elements code, we are bound by the disclosure policy of life$^{\texttt {x}}$, for which nowadays the EM module is not public. Regarding patients’ data, they are bound by an agreement between S. Maria del Carmine Hospital and Politecnico di Milano. To make them available, specific administrative agreements will be needed.

Several further developments will be considered in future works to make our procedure more accurate, efficient, and able to provide more precise clinical information. The previous limitations naturally suggest some of them: the inclusion of the atria, right ventricle, and Purkinje network, and the full automation of the computational pipeline.

Another interesting development of the present study consists in performing, beyond personalized predictive CRT numerical simulations, a statistical analysis of several outputs of CRT numerical experiments in order to provide *a priori* information on the optimality of CRT before the simulation. To do this, we will need to study more cases allowing to provide precise indications on the stimulations regions and delays that could improve CRT, possibly clustering the patients in accordance with their conditions (presence of fibrosis, blocks, etc.).

Also, the proposed framework could be used in future to assess the sensitivity of the CRT outcomes and performance on different degrees of fibrosis distributions, virtually generated in specific cases.

Finally, we mention recent stimulation strategies based on multipoint pacing, for example based on the left ventricular quadripolar lead that allows to stimulate the coronary sinus in different sites, thus improving the contraction syncronym in cases where tissue damages are present (Zanon et al. 2015). Our computational framework could be in principle straightforwardly applied to such scenarios since the sites of stimulation could be easily selected in our code.

### Final remarks

The use of computational methods for the study of the performance of CRT has attracted great interest in the recent years. One of the challenging topic in this field is the optimization of the therapy in terms of electrodes’ positioning and VV delay. The research community has done big improvements in this direction. We (the research community) are still far from providing a routinary tool, possibly implemented in the clinical setting, which could help the clinicians in their decisions, but the improvements made in the recent years by the community are very promising. In this direction, in this work we provided our contribution by introducing for the first time the reconstruction of the epicardial veins, in order to locate the left electrode, and the variation of the right electrode to explore new pacing possibilities.

## Figures and Tables

**Fig. 10:**
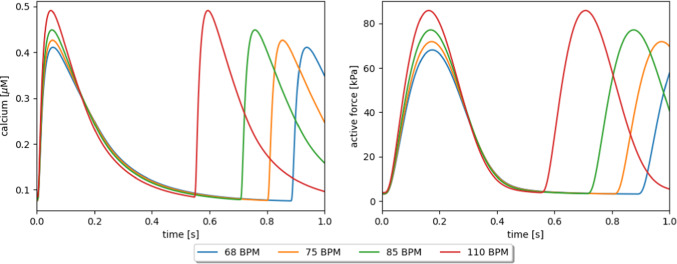
Calcium concentration and active force as the results of single-cell simulations varying the heart rate

## Data Availability

The Finite Elements code, the data for calibration, the input data, the MRI and CT images, the EAMS data, the reconstructed domains and the computational meshes are not public nor available.

## Appendix

### Complete list of quantitative biomarkers results

We report in the three following tables the complete list of results for the biomarkers considered in Tables 5, 6 and 7 for all the virtual scenarios considered in this work.

See (Tables 8, 9 and 10).

**Table 8:** Biomarkers corresponding to all the virtual CRT configurations varying left lead position. See Fig. 7 for the different locations of the left electrode. For CRT scenarios we indicate the percentage of variation of $dP/dt|_{\text {max}}$, *SV* and *SW* with respect to the pre-operative case, whereas for *EF* we indicate the absolute changes. P2, P3, P4: non-fibrotic cases; P6, P8, P11: fibrotic cases

Patient	Location	${dP/dt|}_{{{max}}}$	${ {SV}}$	${{EF}}$	${{SW}}$
		$[{\text {mmHg}\text {s}^{-1}}]$	$[{ml}]$	$[\%]$	$[{\text {mmHg}\,ml}]$
P2	Pre-operative	2252	76	15.1	6300
	*LEAS*	$+7.4\%$	$+11.8\%$	$+1.8$	$+19.4 \%$
	*VEIN 1*	$+8.1\%$	$+14.5\%$	$+2.3$	$+23.5\%$
	*VEIN 2*	$+7.2\%$	$+13.2\%$	$+2.1$	$+22.6\%$
	*VEIN 3*	$+9.9\%$	$+3.9\%$	$+0.6$	$+7.7\%$
	*VEIN 4*	$+12.0\%$	$+11.8\%$	$+1.9$	$+20.1\%$
P3	Pre-operative	3119	112	31.9	11, 600
	*LEAS*	$+13.0\%$	$+8.0\%$	$+2.6$	$+14.8\%$
	*VEIN 1*	$+9.7\%$	$+9.8\%$	$+3.1$	$+16.5\%$
	*VEIN 2*	$+13.9\%$	$+8.0\%$	$+2.7$	$+17.3\%$
	*VEIN 3*	$+2.1\%$	$+2.7\%$	$+1.1$	$+5.5\%$
	*VEIN 4*	$-15.0\%$	$+0.0\%$	$+0.1$	$-0.8\%$
P4	Pre-operative	2278	73	24.5	6800
	*LEAS*	$+6.5\%$	$+7.3\%$	$+1.8$	$+10.8\%$
	*VEIN 1*	$+3.1\%$	$+6.3\%$	$+1.5$	$+10.0\%$
	*VEIN 2*	$+2.8\%$	$+6.2\%$	$+1.5$	$+9.4\%$
	*VEIN 3*	$+3.5\%$	$+4.8\%$	$+1.2$	$+7.8\%$
	*VEIN 4*	$+6.6\%$	$+1.9\%$	$+0.5$	$+3.7\%$
P6	Pre-operative	2186	82	25.9	7000
	*LEAS*	$+3.7\%$	$+11.8\%$	$+3.0$	$+16.9\%$
	*VEIN 1*	$+2.0\%$	$+10.8\%$	$+2.8$	$+15.4\%$
	*VEIN 2*	$-0.3\%$	$+8.5\%$	$+2.2$	$+12.3\%$
	*VEIN 3*	$-2.6\%$	$+5.7\%$	$+1.5$	$+8.0\%$
	*VEIN 4*	$-3.1\%$	$+4.5\%$	$+1.2$	$+6.3\%$
P8	Pre-operative	2731	70	32.9	6700
	*LEAS*	$-7.5\%$	$-1.4\%$	$-0.3$	$-1.6\%$
	*VEIN 1*	$+2.0\%$	$+4.2\%$	$+1.4$	$+6.6\%$
	*VEIN 2*	$-2.20\%$	$+2.8\%$	$+1.0$	$+4.0\%$
	*VEIN 3*	$-1.9\%$	$+0.0\%$	$-0.2$	$-0.6\%$
	*VEIN 4*	$-3.0\%$	$-4.2\%$	$-1.6$	$-6.4\%$
P11	Pre-operative	2460	58	25.0	4900
	*LEAS*	$+0.7\%$	$+10.3\%$	$+2.6$	$+15.6\%$
	*VEIN 1*	$-2.0\%$	$+1.7\%$	$+0.6$	$+3.9\%$
	*VEIN 2*	$-2.6\%$	$+5.2\%$	$+1.6$	$+9.5\%$
	*VEIN 3*	$-0.7\%$	$+0.6\%$	$+2.4$	$+15.2\%$
	*VEIN 4*	$+1.5\%$	$+6.9\%$	$+1.9$	$+12.4\%$

**Table 9:** Biomarkers corresponding to all the virtual CRT configurations varying Ventriculo-Ventricular Delay (*VVD*) with the left electrode positioned at LEAS. Positive values of *VVD* mean that the right stimulation occurs before the left one. For CRT scenarios we indicate the percentage of variation of $dP/dt|_{\text {max}}$, *SV* and *SW* with respect to the pre-operative case, whereas for *EF* we indicate the absolute changes. P2, P3, P4: non-fibrotic cases; P6, P8, P11: fibrotic cases

Patient	Delay	${dP/dt|}_{{ {max}}}$	${{SV}}$	${ {EF}}$	${{SW}}$
	[ms]	$[{\text {mmHg}\text {s}^{-1}}]$	$[{ml}]$	$[\%]$	$[{\text {mmHg}\,ml}]$
P2	*Pre-operative*	2252	76	15.1	6300
	$-30$	$+4.3\%$	$+7.9\%$	$+1.4$	$+13.0 \%$
	$-15$	$+5.2\%$	$+10.5\%$	$+1.6$	$+17.0\%$
	0	$+7.4\%$	$+11.8\%$	$+1.8$	$+19.0\%$
	15	$+9.3\%$	$+11.8\%$	$+1.8$	$+20.0\%$
	30	$+11.3\%$	$+10.5\%$	$+1.7$	$+18.0\%$
P3	Pre-operative	3119	112	31.9	11, 600
	$-30$	$+2.9\%$	$+5.4\%$	$+1.7$	$+9.0\%$
	$-15$	$+7.0\%$	$+7.1\%$	$+2.3$	$+12.2\%$
	0	$+13.0\%$	$+8.0\%$	$+2.6$	$+14.8\%$
	15	$+17.7\%$	$+8.9\%$	$+2.9$	$+16.2\%$
	30	$+24.9\%$	$+8.9\%$	$+2.8$	$+16.6\%$
P4	Pre-operative	2278	73	24.5	6800
	$-30$	$-0.1\%$	$+5.3\%$	$+1.3$	$+8.0\%$
	$-15$	$+1.2\%$	$+6.2\%$	$+1.5$	$+9.8\%$
	0	$+6.5\%$	$+7.3\%$	$+1.8$	$+10.8\%$
	15	$+9.8\%$	$+7.6\%$	$+1.8$	$+10.9\%$
	30	$+9.1\%$	$+6.9\%$	$+1.7$	$+9.8\%$
**P6**	Pre-operative	2186	82	25.9	7000
	$-30$	$+1.6\%$	$+10.9\%$	$+2.8$	$+15.2$
	$-15$	$+2.4\%$	$+11.5\%$	$+3.0$	$+16.6\%$
	0	$+3.7\%$	$+11.8\%$	$+3.0$	$+16.9\%$
	15	$+5.1\%$	$+10.6\%$	$+2.7$	$+15.4\%$
	30	$+6.7\%$	$+9.7\%$	$+2.5$	$+13.4\%$
P8	Pre-operative	2731	70	32.9	6700
	$-30$	$-3.1\%$	$+1.4\%$	$+0.6$	$+1.3\%$
	$-15$	$-0.9\%$	$+2.8\%$	$+1.1$	$+6.9\%$
	0	$+2.0\%$	$4.2\%$	$+1.4$	$+6.6\%$
	15	$+3.0\%$	$+4.2\%$	$+1.5$	$+6.9\%$
	30	$+3.2\%$	$+2.8\%$	$+1.1$	$+5.4\%$
P11	Pre-operative	2460	58	25.0	4900
	$-30$	$-0.1\%$	$+5.2\%$	$+1.5$	$+8.6\%$
	$-15$	$+0.5\%$	$+6.9\%$	$+2.1$	$+12.4\%$
	0	$+0.7\%$	$+10.3\%$	$+2.6$	$+15.6\%$
	15	$-0.9\%$	$+8.6\%$	$+2.2$	$+13.2\%$
	30	$-1.7\%$	$+6.9\%$	$+1.7$	$+9.0\%$

**Table 10:** Biomarkers corresponding to all the virtual CRT configurations varying right lead position, with LEAS choice for the left one and $VVD = 0$. *RE* position indicated the apical position used in the numerical experiments reported in Sects. 3.3 and 3.4. See Fig. 9 for the different locations of the right electrode. For CRT scenarios we indicate the percentage of variation of $dP/dt|_{\text {max}}$, *SV* and *SW* with respect to the pre-operative case, whereas for *EF* we indicate the absolute changes. P2, P3, P4: non-fibrotic cases; P6, P8, P11: fibrotic cases

Patient	Location	${dP/dt|}_{{ {max}}}$	${ {SV}}$	${{EF}}$	${{SW}}$
		$[{\text {mmHg}\text {s}^{-1}}]$	$[{ml}]$	$[\%]$	$[{\text {mmHg}\,ml}]$
P2	Pre-operative	2252	76	15.1	6300
	*RE*	$+7.4\%$	$+11.8\%$	$+1.8$	$+19.4 \%$
	*RE 1*	$-0.8\%$	$+14.5\%$	$+2.2$	$+23.5\%$
	*RE 2*	$-0.4\%$	$+18.4\%$	$+2.8$	$+30.1\%$
	*RE 3*	$+3.5\%$	$+19.7\%$	$+3.1$	$+33.4\%$
P3	Pre-operative	3119	112	31.9	11, 600
	*RE*	$+13.0\%$	$+8.0\%$	$+2.6$	$+14.8 \%$
	*RE 1*	$-0.5\%$	$+3.6\%$	$+1.3$	$+7.0\%$
	*RE 2*	$+11.0\%$	$+9.8\%$	$+3.3$	$+17.8\%$
	*RE 3*	$+14.5\%$	$+12.5\%$	$+4.2$	$+22.3\%$
P4	Pre-operative	2278	73	24.5	6800
	*RE*	$+6.5\%$	$+7.3\%$	$+1.8$	$+10.8\%$
	*RE 1*	$+5.8\%$	$+5.6\%$	$+1.4$	$+9.5\%$
	*RE 2*	$+7.9\%$	$+12.2\%$	$+3.0$	$+19.3\%$
	*RE 3*	$+4.9\%$	$+13.6\%$	$+3.3$	$+20.1\%$
P6	Pre-operative	2186	82	25.9	7000
	*RE*	$+3.7\%$	$+11.8\%$	$+3.0$	$+16.9\%$
	*RE 1*	$+2.2\%$	$-0.1\%$	$+0$	$+1.1\%$
	*RE 2*	$+5.6\%$	$+4.8\%$	$+1.2$	$+8.9\%$
	*RE 3*	$+7.9\%$	$+12.5\%$	$+3.2$	$+19.3\%$
P8	Pre-operative	2731	71	33.2	6700
	*RE*	$-7.5\%$	$-1.4\%$	$-0.3$	$-1.6\%$
	*RE 1*	$-5.7\%$	$+7.0\%$	$+2.3$	$+9.7\%$
	*RE 2*	$-5.1\%$	$+8.5\%$	$+2.6$	$+11.3\%$
	*RE 3*	$-6.7\%$	$+5.6\%$	1.6	$+7.2\%$
P11	Pre-operative	2460	58	25.0	4900
	*RE*	$+0.7\%$	$+10.3\%$	$+2.6$	$+15.6\%$
	*RE 1*	$+3.0\%$	$+6.9\%$	$+1.9$	$+12.6\%$
	*RE 2*	$+2.6\%$	$+12.1\%$	$+3.1$	$+19.6\%$

### A note on the mechanical model

In Fig. 10, we report results of single-cell simulations by coupling the ToR-ORd ionic model (Tomek et al. 2019) with the *RDQ20-MF* model active force generation model (Regazzoni et al. 2020), by considering four increasing values of heart rates. The simulations are run until a limit cycle is reached, and the time variable is then shifted to zero to enable comparison. Notice that, thanks to the capability of the ToR-ORd model to reproduce the restitution curve of cytosolic calcium, higher heart rates correspond to higher peaks in calcium concentration. In turn, thanks to the capability of the RDQ20-MF model to reproduce the force-calcium dependency, higher calcium values translate into higher generated force. Overall, the coupled model captures the dependence of contractility upon variations in the heart rate.

## Footnotes

1 https://lifex.gitlab.io/lifex.

2 https://github.com/FrancescoRegazzoni/cardioemulator
